# Supplementary material for: Spatio-temporal modeling of high-throughput multispectral aerial images improves agronomic trait genomic prediction in hybrid maize
Source: Genetics. 2024 Mar 12;227(1):iyae037. doi: 10.1093/genetics/iyae037 (PMC11075545; doi:10.1093/genetics/iyae037)
Supplement: iyae037_Supplementary_Data [file iyae037_supplementary_data.zip › File_S1_GENETICS-2024-306855.docx]

## File S1: Simulations

The first simulation process, the linear process, followed an evaluation of


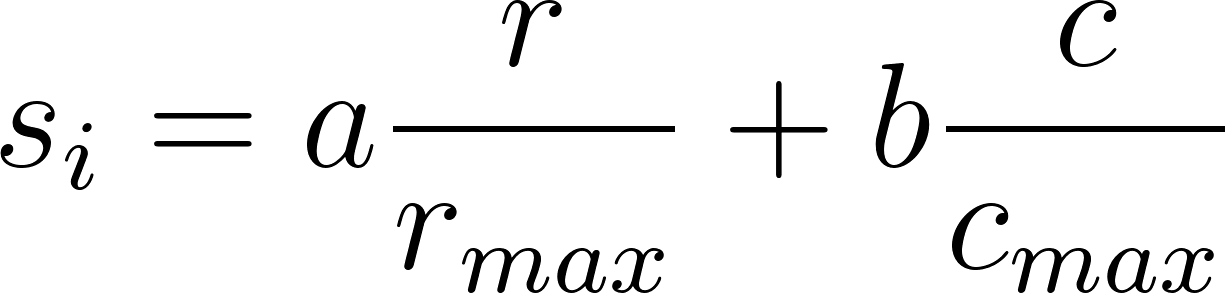
 (Simulation 1)

where
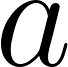
 and
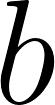
 were randomly assigned numbers between 0 and 1,
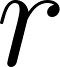
 and
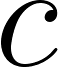
 were the current plot’s row and column position in the field, and
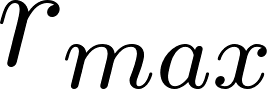
 and
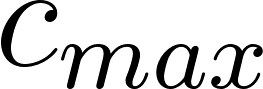
 were the maximum row and column numbers in the field, respectively. The variable
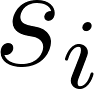
 was the simulated environmental effect for a specific experimental plot. Simulation 1 resulted in a constant gradient traversing the field, which increased as the row and column position increased. The second simulation process, the one-dimensional normal (1D-N) process, followed an evaluation of the univariate normal distribution equation below.


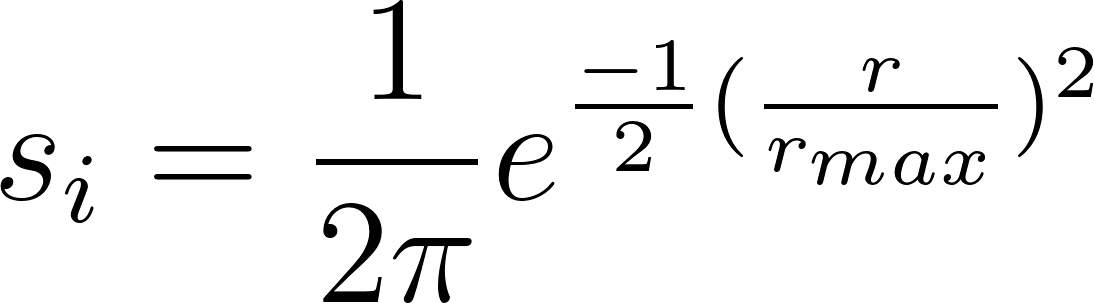
(Simulation 2)

Simulation 2 resulted in a normal gradient which was constant across columns, but increased across the rows. The third simulation process, the two-dimensional normal (2D-N) process, followed an evaluation of the bivariate normal distribution equation


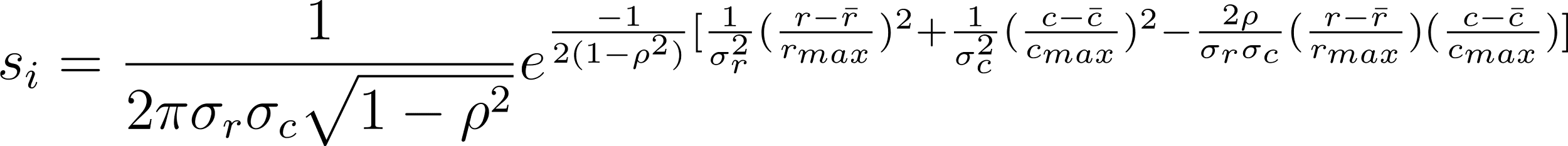
(Simulation 3)

where
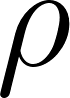
 was a randomly assigned correlation between row number
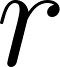
 and column number
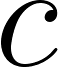
,
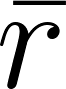
 and
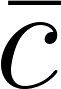
 denoted the mean row and column numbers, respectively, and
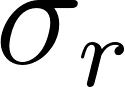
 and
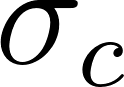
 denoted the standard deviations among the rows and columns, respectively. Simulation 3 resulted in a peak in the center of the field with normally distributed gradients decreasing across the rows and columns; the skew in the row and column gradients was controlled by
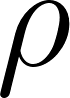
. The fourth simulation process, the separable autoregressive process (AR1xAR1), followed a multivariate normal distribution


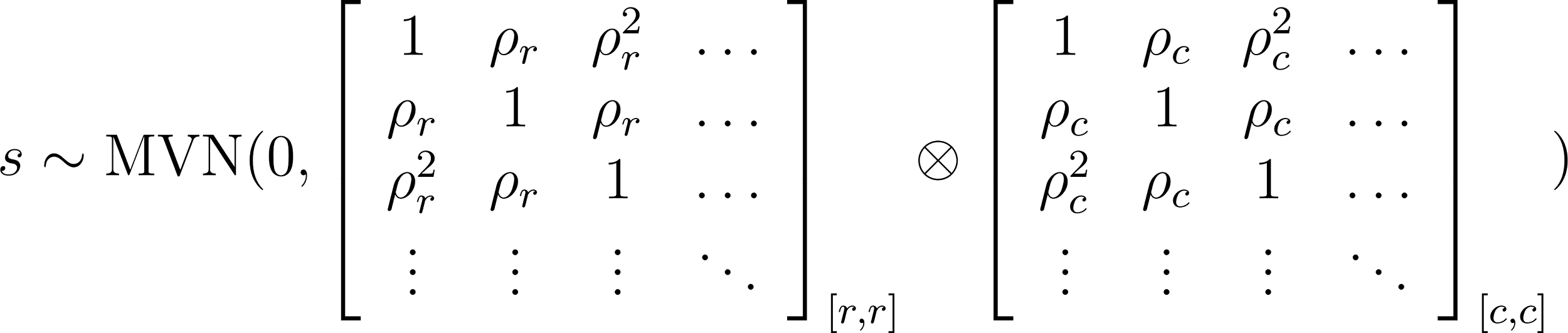
(Simulation 4)

where
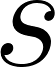
 was a vector of simulated values, and
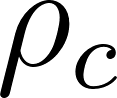
 and
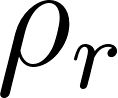
 were randomly assigned correlations among the rows and columns, respectively. Simulation 4 explicitly defined a decay in the correlation between experimental plots based on their proximity on a unit-by-unit basis (e.g.
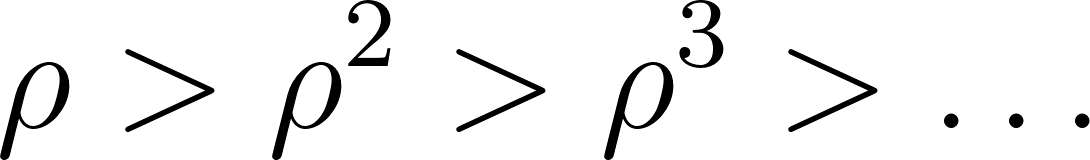
). The multivariate normal distribution was implemented using the MASS package (Venables and Ripley 2002). The fifth simulation process, the random process, followed


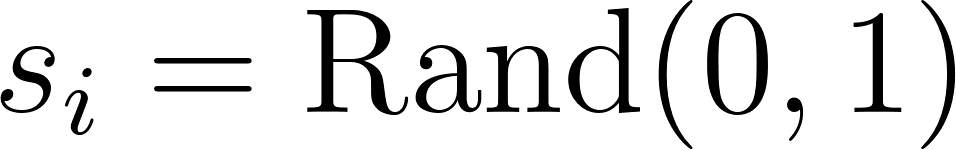
 (Simulation 5)

and produced random values between 0 and 1. Simulation 5 was used as a control to determine the effect of random noise. The sixth simulation process, the real data (RD) process, was intended to use the collected soil EC data directly


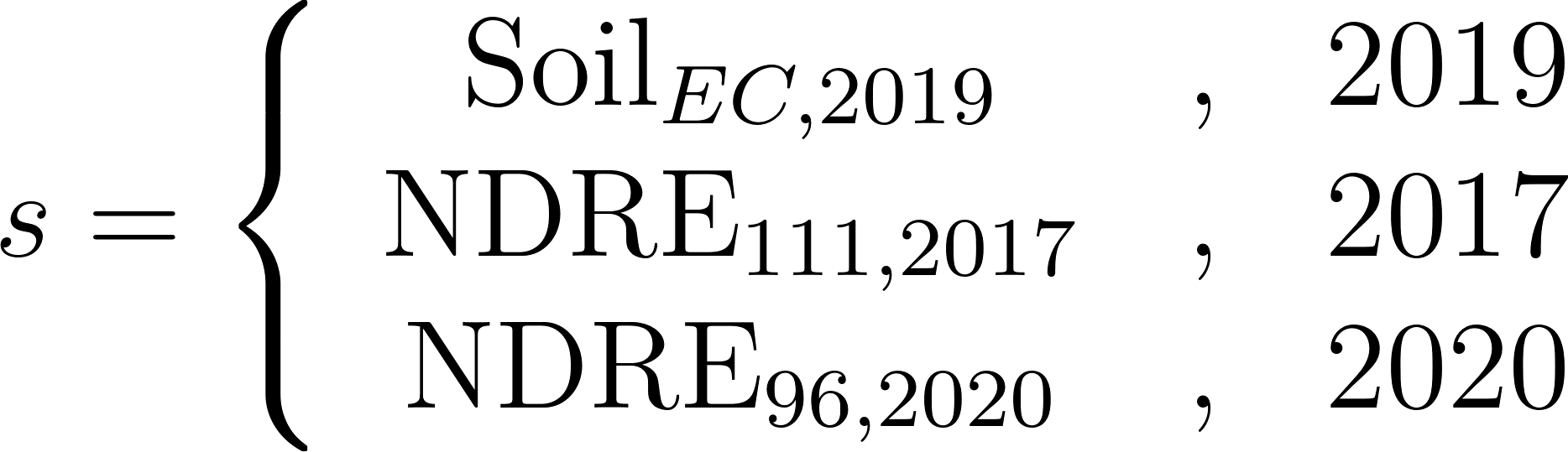
 (Simulation 6)

where the vector of simulated values
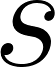
 was the soil EC data from 2019. In 2017 and 2020 soil data was not available, so NDRE at 111 and 96 days after planting, respectively, was used instead. It was important to use a measurement with no missing data, therefore, the HTP measurement of NDRE was suitable.

For each of the six simulations described, three approaches were explored for generating the simulated field effect across time. The first approach defined the simulated field effect as constant through time. This can be written as
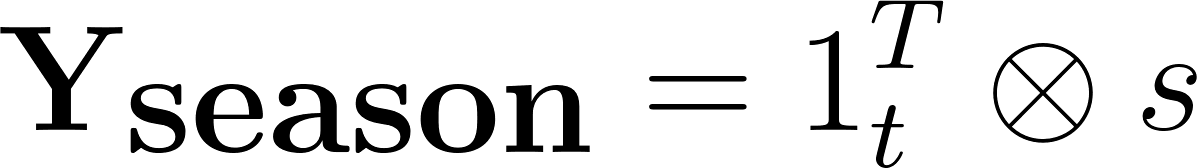
, where
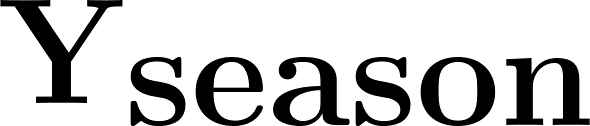
 was a matrix representing all simulated values across the season,
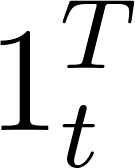
 was a transposed vector of ones with length equal to the number of time points
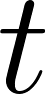
, and
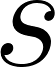
 contained the simulated values from one of the six aforementioned simulation processes. Figure S5 illustrated heatmaps for all six simulation processes, constant over 12 timepoints. The second and third approaches generated simulated values which were 90% and 75%, respectively, correlated through time. This was accomplished by specifying a correlation structure
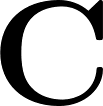
 between time points as
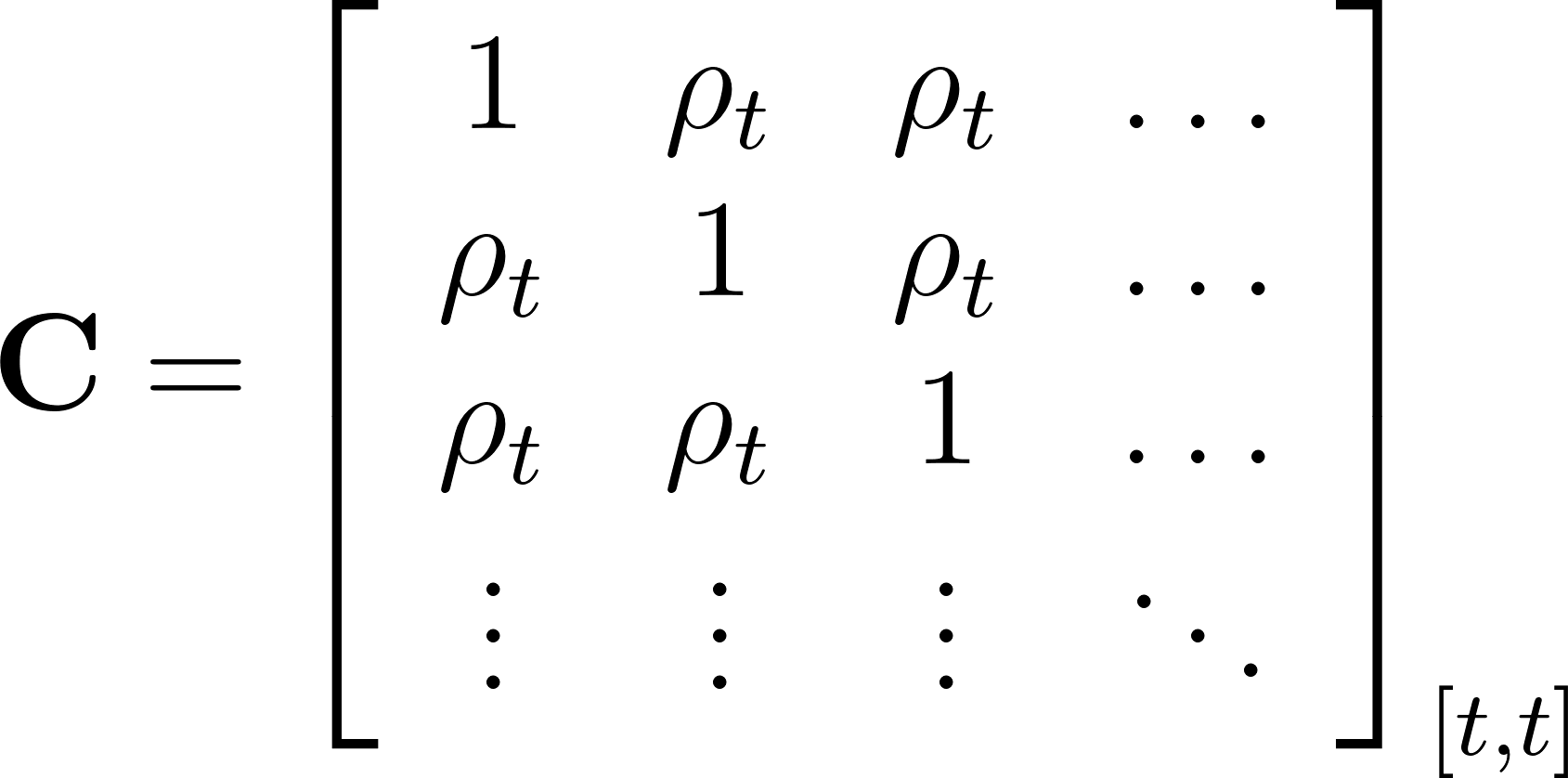
, where
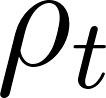
 was the correlation between time points, set to either 0.75 or 0.90, and the dimensions of
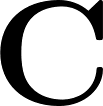
 were
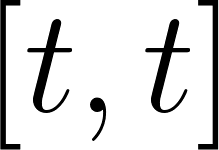
 to denote the number of time points. Simulated correlated values
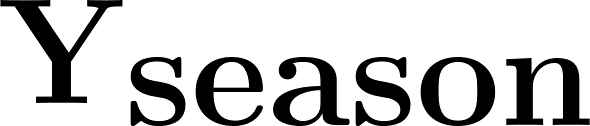
 were generated across the growing season by first taking the Cholesky decomposition,
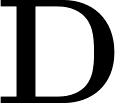
, of
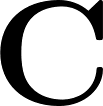
 as
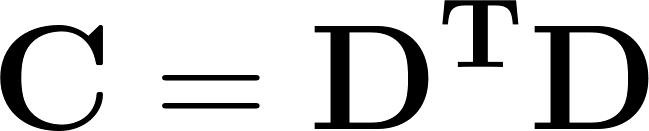
. Then,
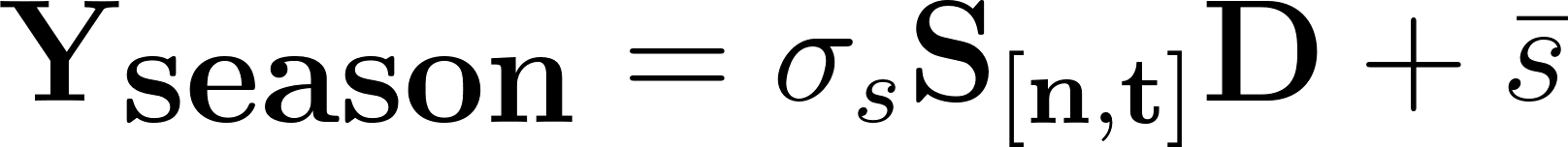
 was defined, where
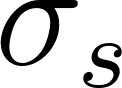
 and
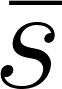
 are the standard deviation and mean of the target simulated values
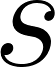
, respectively. The matrix
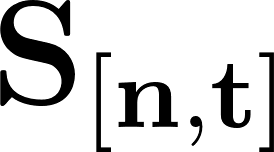
 contained the normalized simulated values
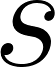
 in the first column, followed by columns initialized by a unit normal distribution resulting in
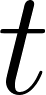
 columns. Figure S6 illustrated heatmaps for all six simulation processes over 12 time points which were 90% correlated to each other.
