# Supplementary material for: Spatio-temporal modeling of high-throughput multispectral aerial images improves agronomic trait genomic prediction in hybrid maize
Source: Genetics. 2024 Mar 12;227(1):iyae037. doi: 10.1093/genetics/iyae037 (PMC11075545; doi:10.1093/genetics/iyae037)
Supplement: iyae037_Supplementary_Data [file iyae037_supplementary_data.zip › File_S2_GENETICS-2024-306855.docx]

## File S2: Spatial Mixed Models

The 2DSpl method created
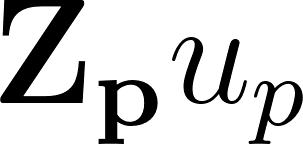
 as a function of the row (
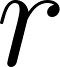
) and column (
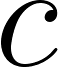
) position of the plot in the field and can be written as


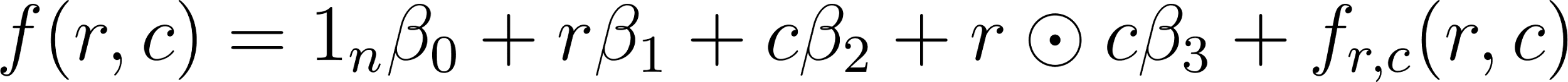
 (Equation S1)

where
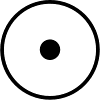
 is the vector product and
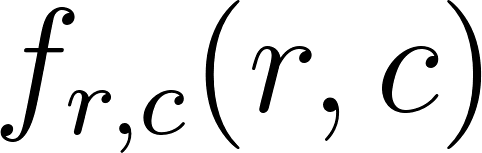
 is a smoothing function (Rodríguez-Álvarez et al. 2018). The 2DSpl models were fitted using Sommer in R (R 3.6.3, Sommer 4.1.3) (Covarrubias-Pazaran 2016).

The AR1 method explicitly defined a separable autoregressive covariance structure. In the single-trait case, named AR1U, the spatial variance was defined as


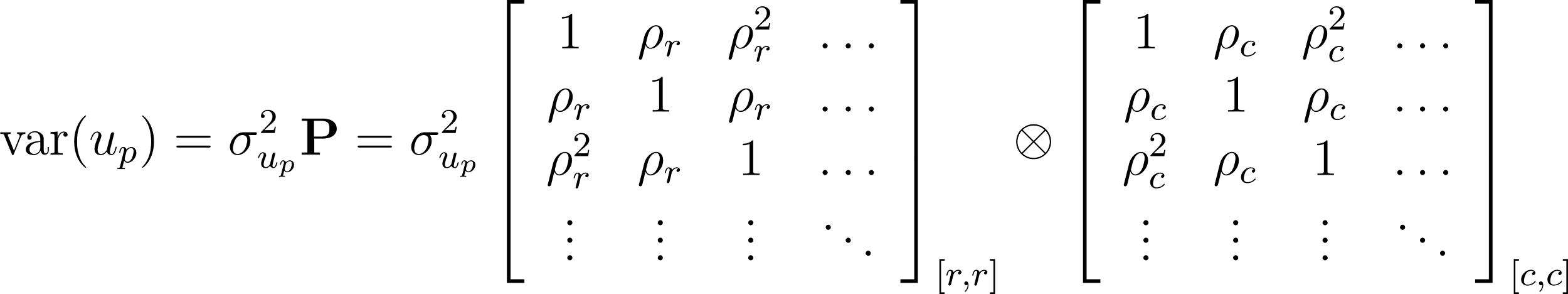


(Equation S2)

where
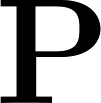
 was a shorthand for the illustrated separable autoregressive structure,
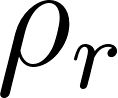
 and
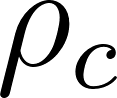
 were correlations among rows and columns, respectively, and
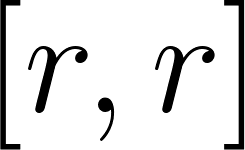
 and
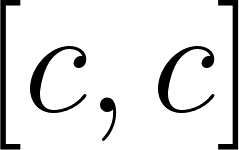
 indicated the dimensions for the matrices as the total number of rows and columns, respectively. With the AR1 model, experimental plots which were farther away had correlations which decreased exponentially on a unit-by-unit basis (e.g.
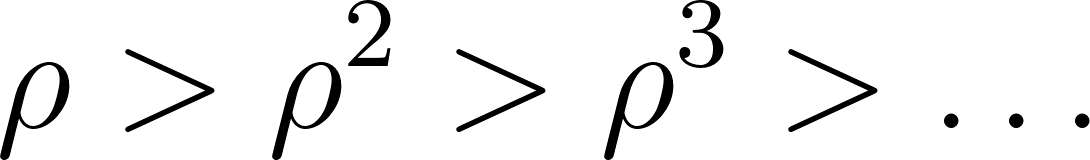
). In the single-trait-repeated case, named AR1M, the variance of the random spatial effect was written as


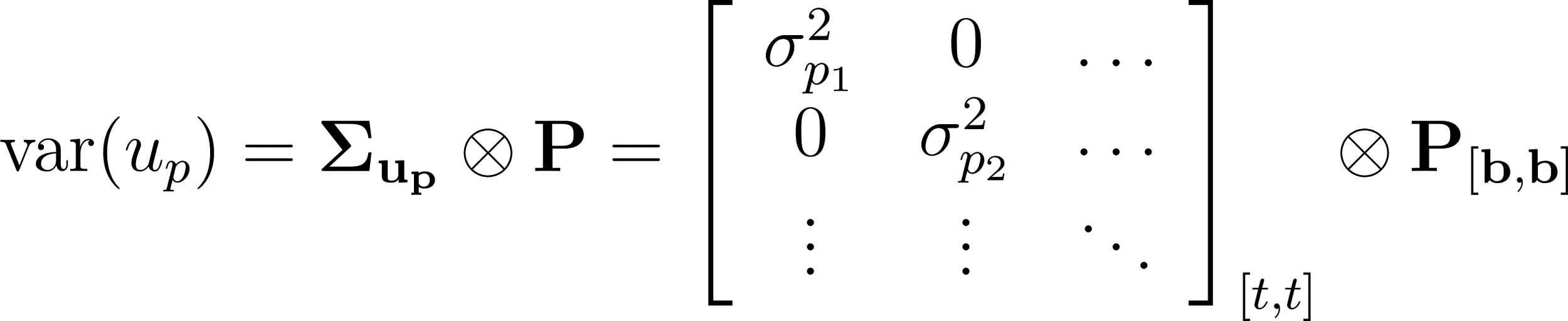
 (Equation S3)

where the diagonal matrix
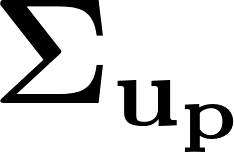
 was of order
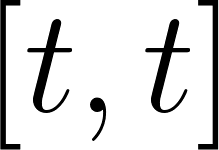
 denoting the number of time points and the matrix
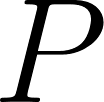
 was the same as in the single-trait case. The AR1 methods were fitted using ASReml-R (R 3.6.3, ASReml-R 4.1.0.126) (A. R. Gilmour et al. 2002). In the tested single-trait and single-trait-repeated spatial models, the random residual error,
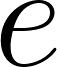
, followed
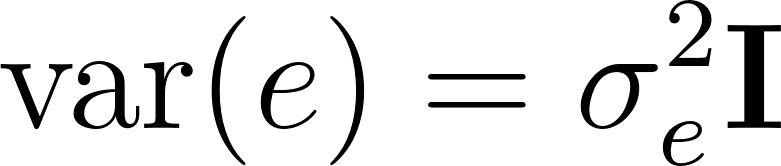
 and
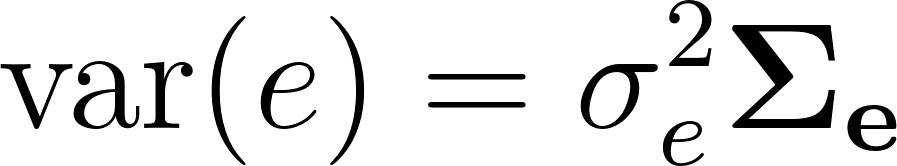
, respectively, where
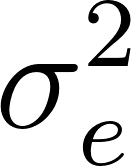
 was the error variance and
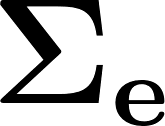
 was unstructured.
