# Supplementary material for: Spatio-temporal modeling of high-throughput multispectral aerial images improves agronomic trait genomic prediction in hybrid maize
Source: Genetics. 2024 Mar 12;227(1):iyae037. doi: 10.1093/genetics/iyae037 (PMC11075545; doi:10.1093/genetics/iyae037)
Supplement: iyae037_Supplementary_Data [file iyae037_supplementary_data.zip › Supplemental_Figures_GENETICS-2024-306855.docx]

## Supplemental Figures


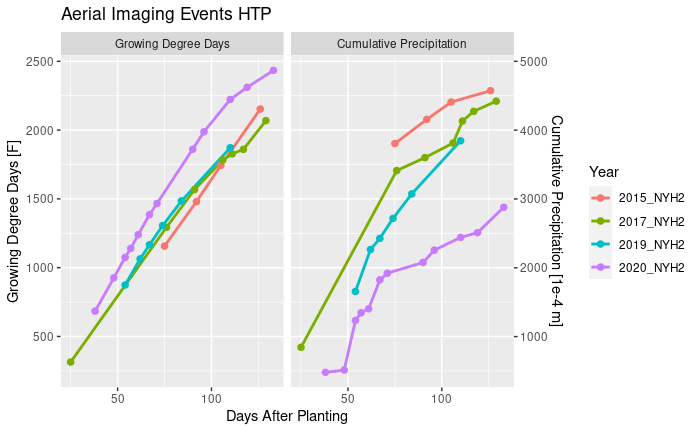


Figure S1: Aerial imaging events with HTP extracted for the hybrid maize experiments in 2015, 2017, 2019, and 2020. Growing degree days (GDD) and cumulative precipitation (CP) were plotted against the days after planting (DAP) of the imaging events. All field experiments were located in Musgrave Research Farm, though planted in distinct fields, and weather data was sourced for the GHCND:USC00300331 ground station via the NOAA NCEI NCDC database.


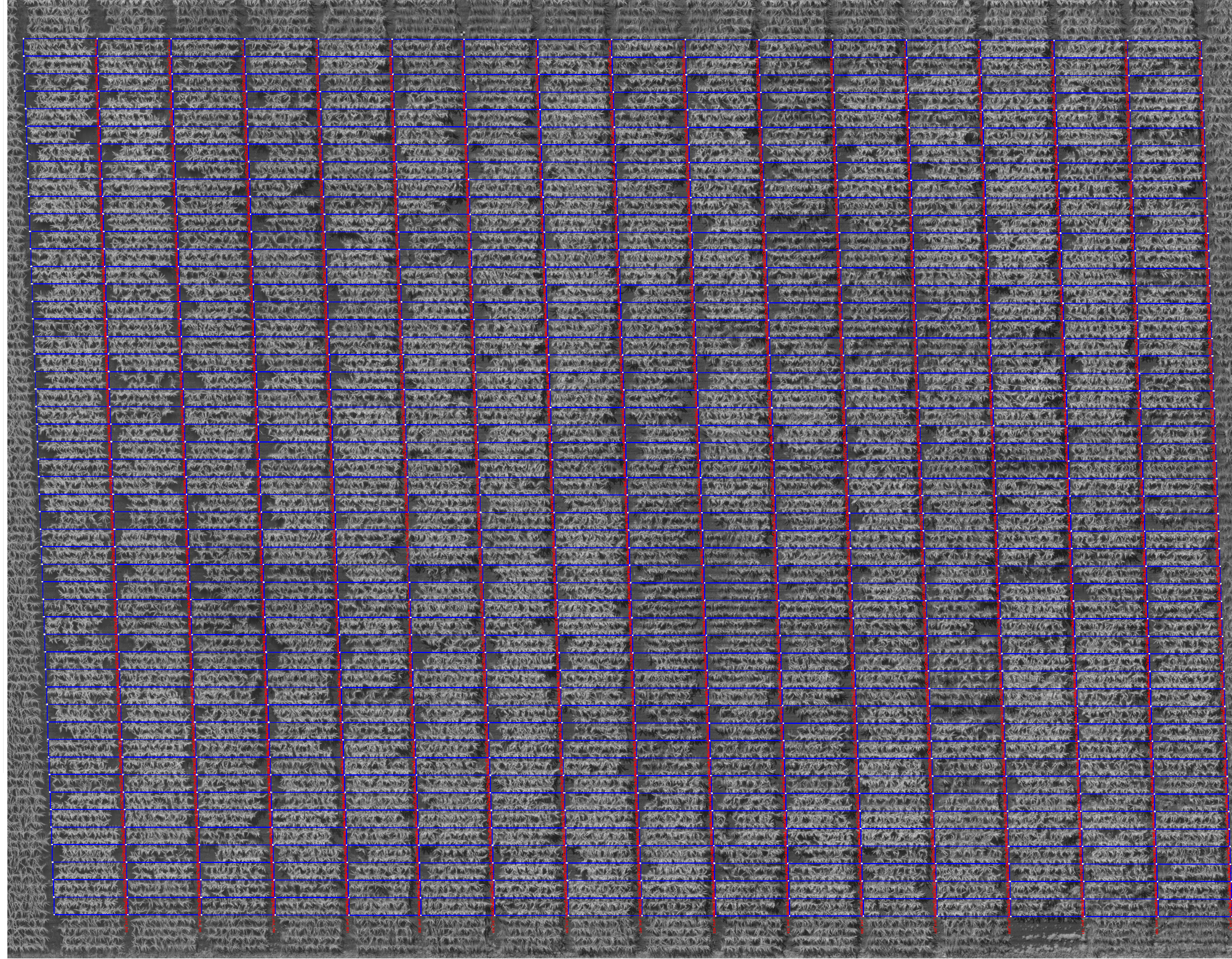


Figure S2: Illustrated is a representative reflectance orthophotomosaic raster image. This image was of 2019_NYH2 on Aug 15, 2019 and was in the near-infrared (NIR) spectra. The original image had dimensions of 6488 by 4987 pixels allowing about 1cm per pixel resolution and was available from <https://imagebreed.org/data/images/image_files/26/05/f9/3e/ab9b340016a1db73f9c743cf/imagegoCo.png>. The overlaid blue polygons represented plot-polygons drawn in ImageBreed to segment plot-images for high-throughput phenotype (HTP) extraction.


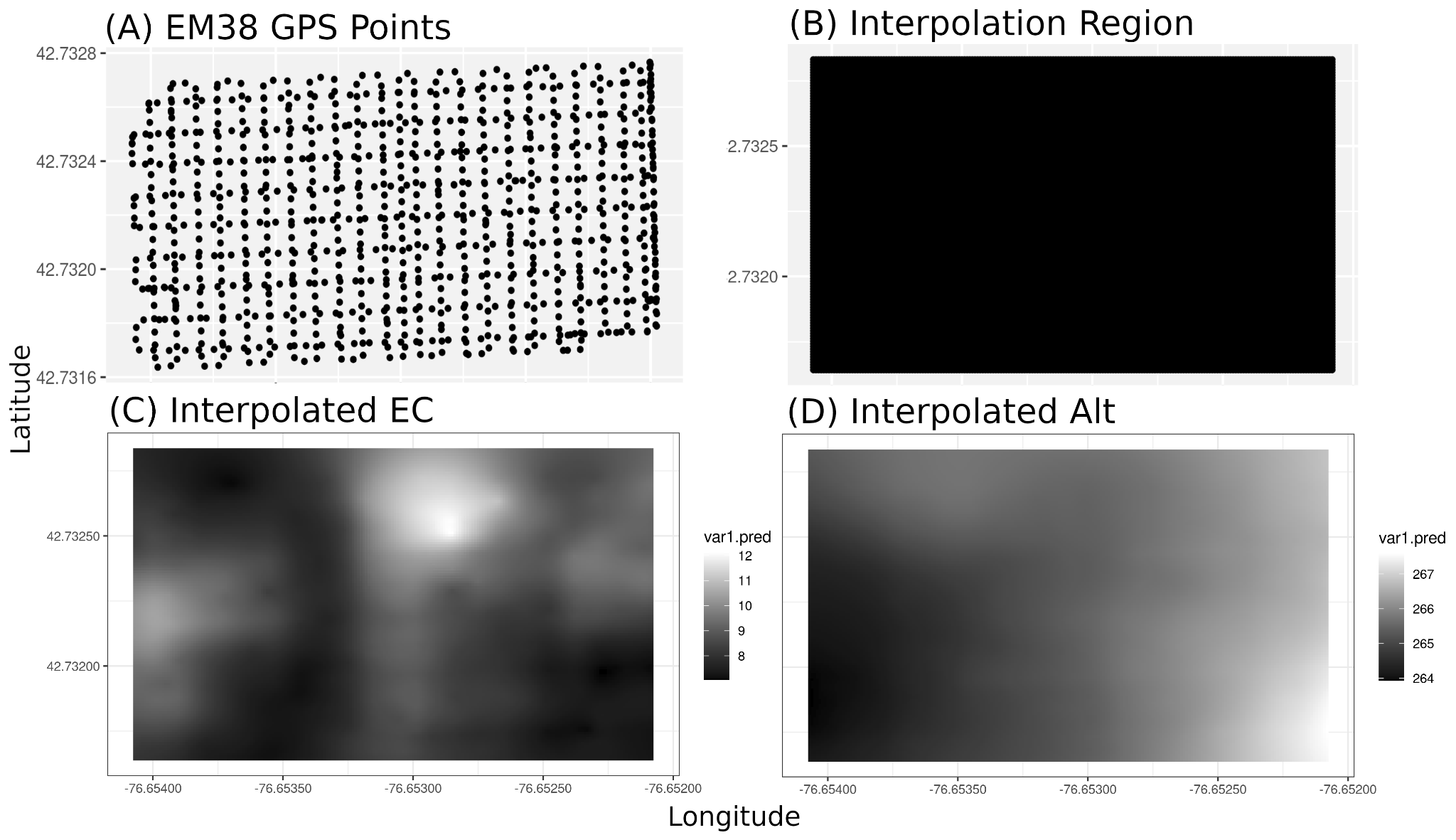


Figure S3: Illustrated are the 2019_NYH2 soil survey interpolation maps. (A) illustrated the raw EM38 soil survey GPS data collected using a dual-serpentine path. (B) illustrated the region to interpolate into with a 0.00001 WGS84 resolution across 200 by 120 cells. Finally, (C) and (D) showed the soil EC and altitude, respectively, interpolated across the field using ordinary Kriging.


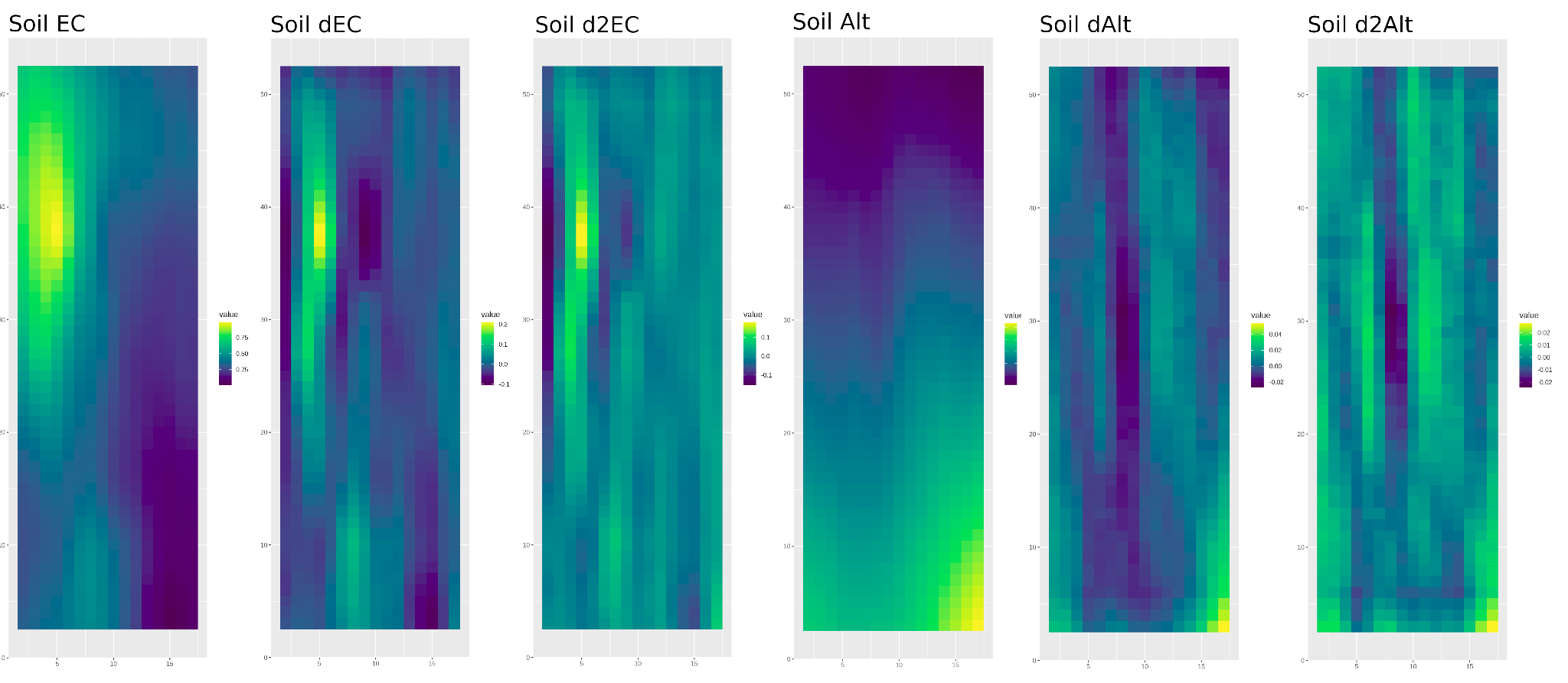


Figure S4: Soil EC and altitude was extracted for the experimental plots in 2019_NYH2. First and second two-dimensional numerical derivatives were computed. The heatmaps illustrated are of these soil measurements for the 800 experimental plots across 16 columns and 50 rows.


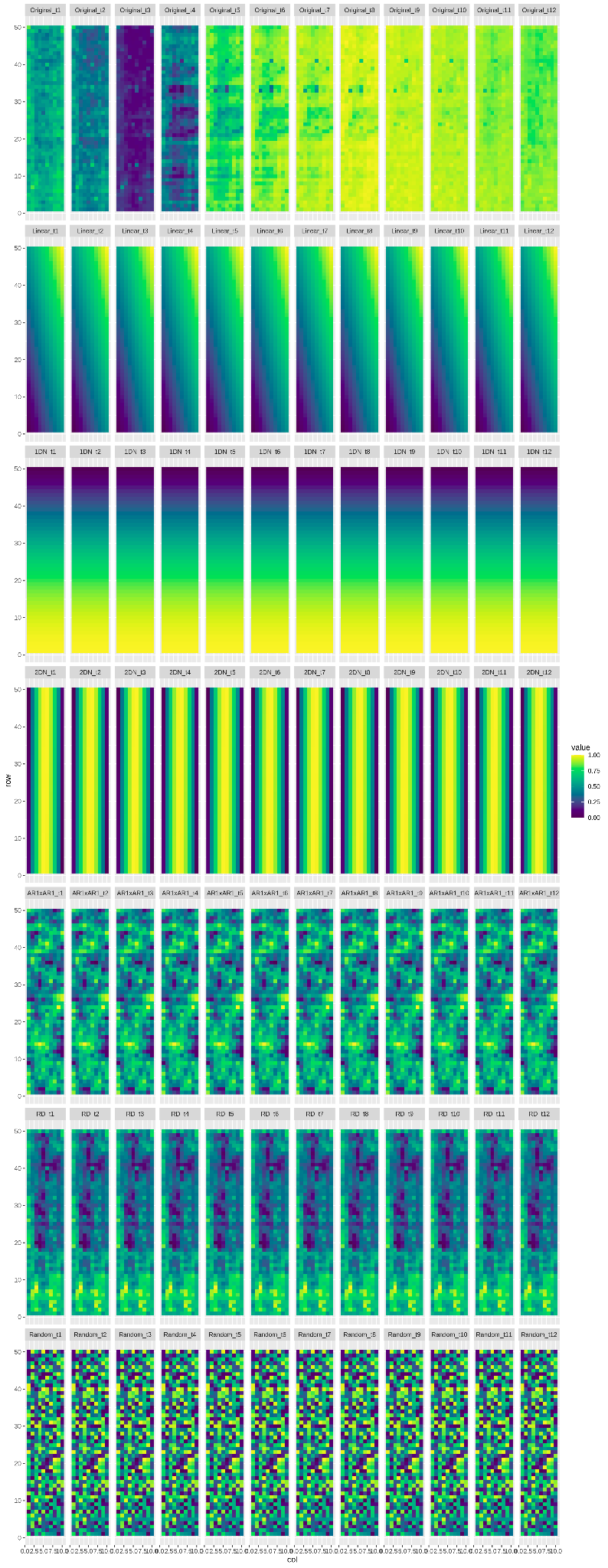


Figure S5: Illustrated are example heatmaps of the six simulation processes, constant through time. At the top, the actual 2020_NYH2 NDVI phenotype across the 12 imaging events was shown, followed by: the linear simulation (Simulation 1), the 1D-N (Simulation 2), the 2D-N (Simulation 3), the separable autoregressive (Simulation 4), the random (Simulation 5), and the real data (Simulation 6) processes, sequentially.


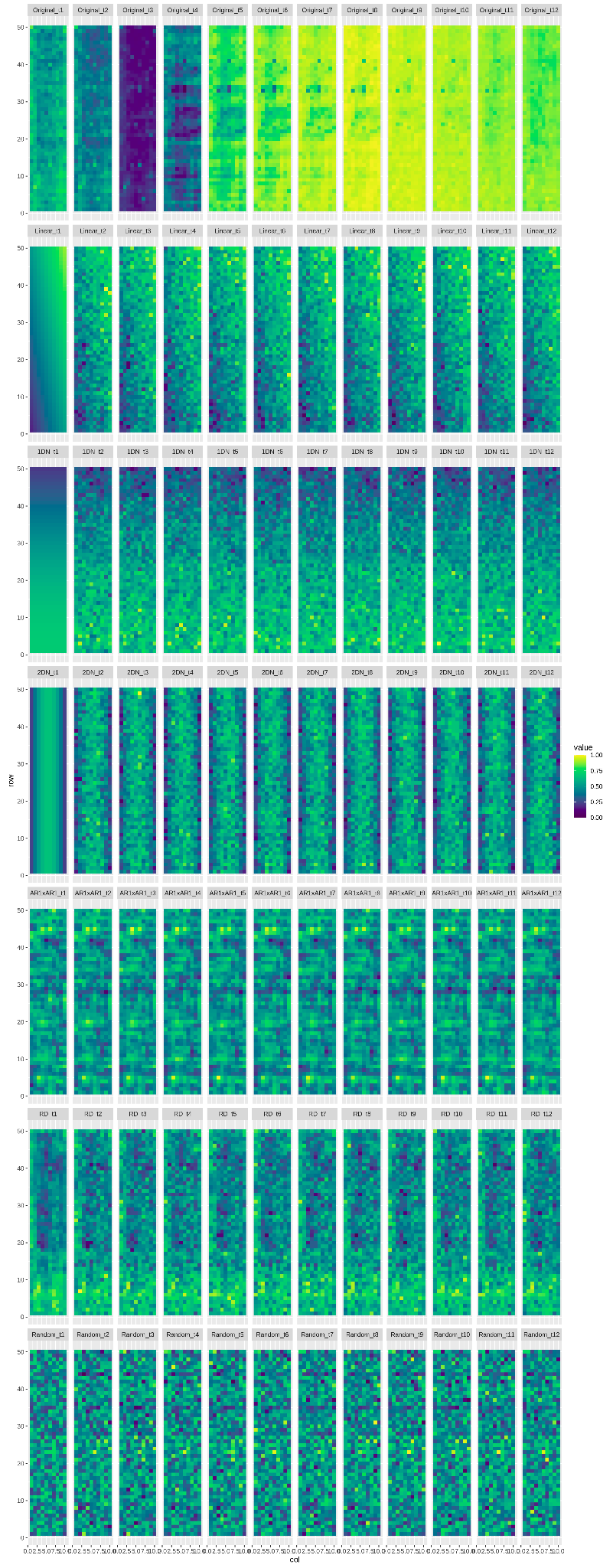


Figure S6: Illustrated are example heatmaps of the six simulation processes, 90% correlated across time. At the top, the 2020_NYH2 NDVI phenotype across the 12 imaging events was shown, followed by: the linear simulation (Simulation 1), the 1D-N (Simulation 2), the 2D-N (Simulation 3), the separable autoregressive (Simulation 4), the random (Simulation 5), and the real data (Simulation 6) processes, sequentially.


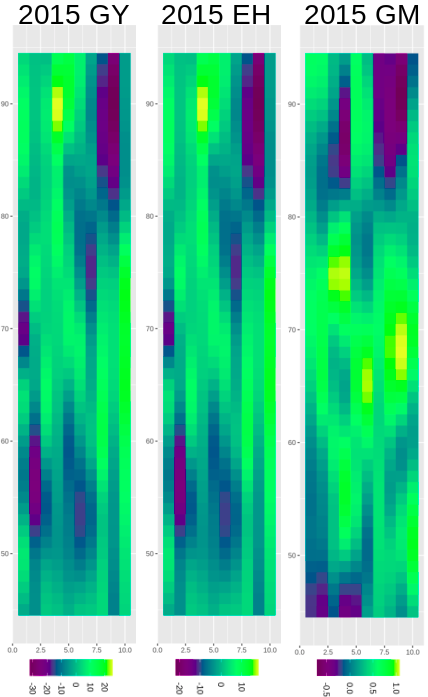


Figure S7: Illustrated are the two-dimensional spline (2DSpl) spatial effects for grain yield (GY), grain moisture (GM), and ear height (EH) in the 2015_NYH2 field experiment. Spatial effects are drawn over the 500 experimental plots across 10 rows and 50 columns.


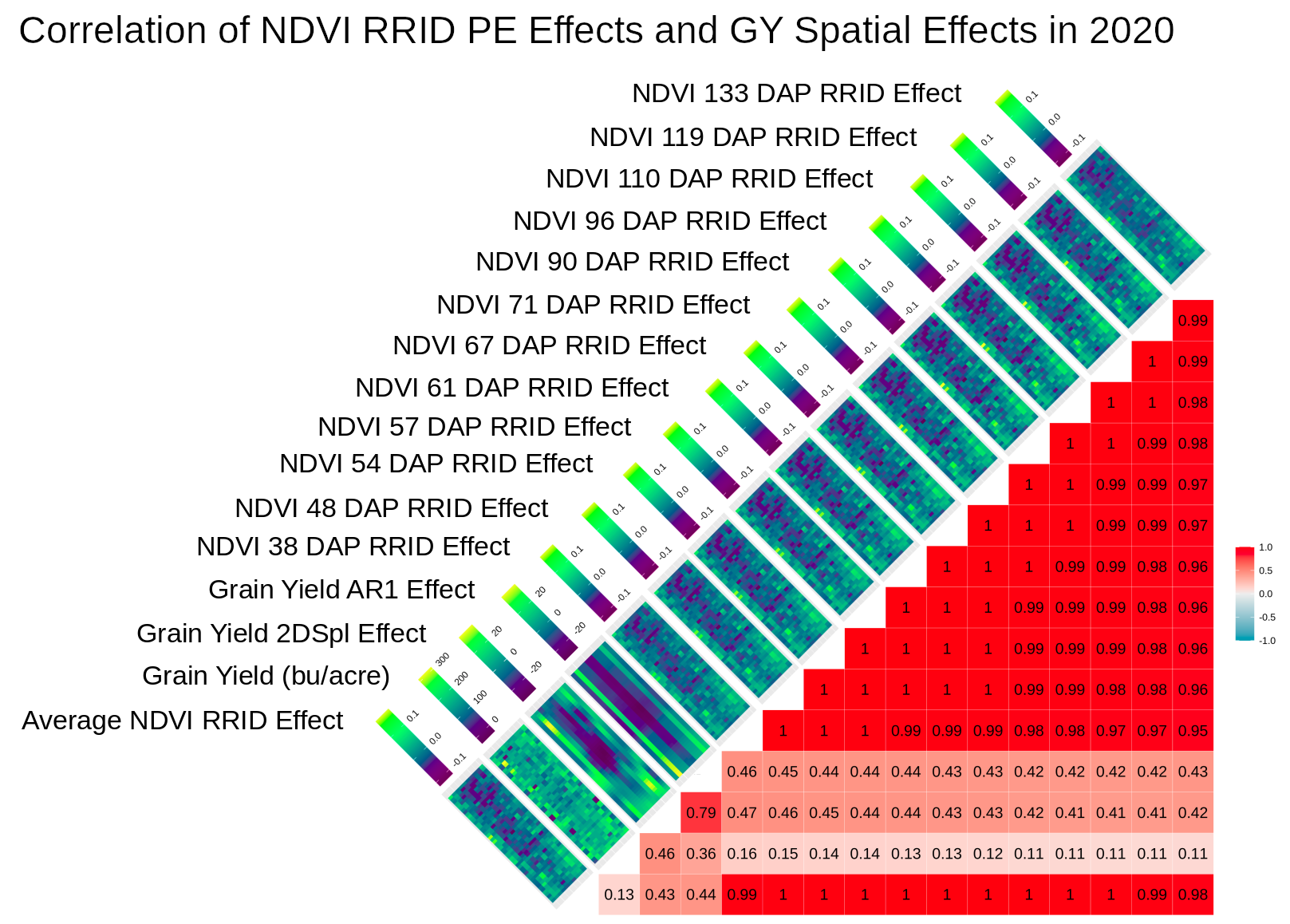


Figure S8: The NDVI RRID PE effects for 12 time points across the 2020_NYH2 growing season correlated with grain yield (GY) and the 2DSpl and AR1 spatial effects of GY. The NDVI PE and the GY spatial effects correlate at a value of 0.5 at 38 days after planting (DAP), and correlate above 0.4 across the growing season.


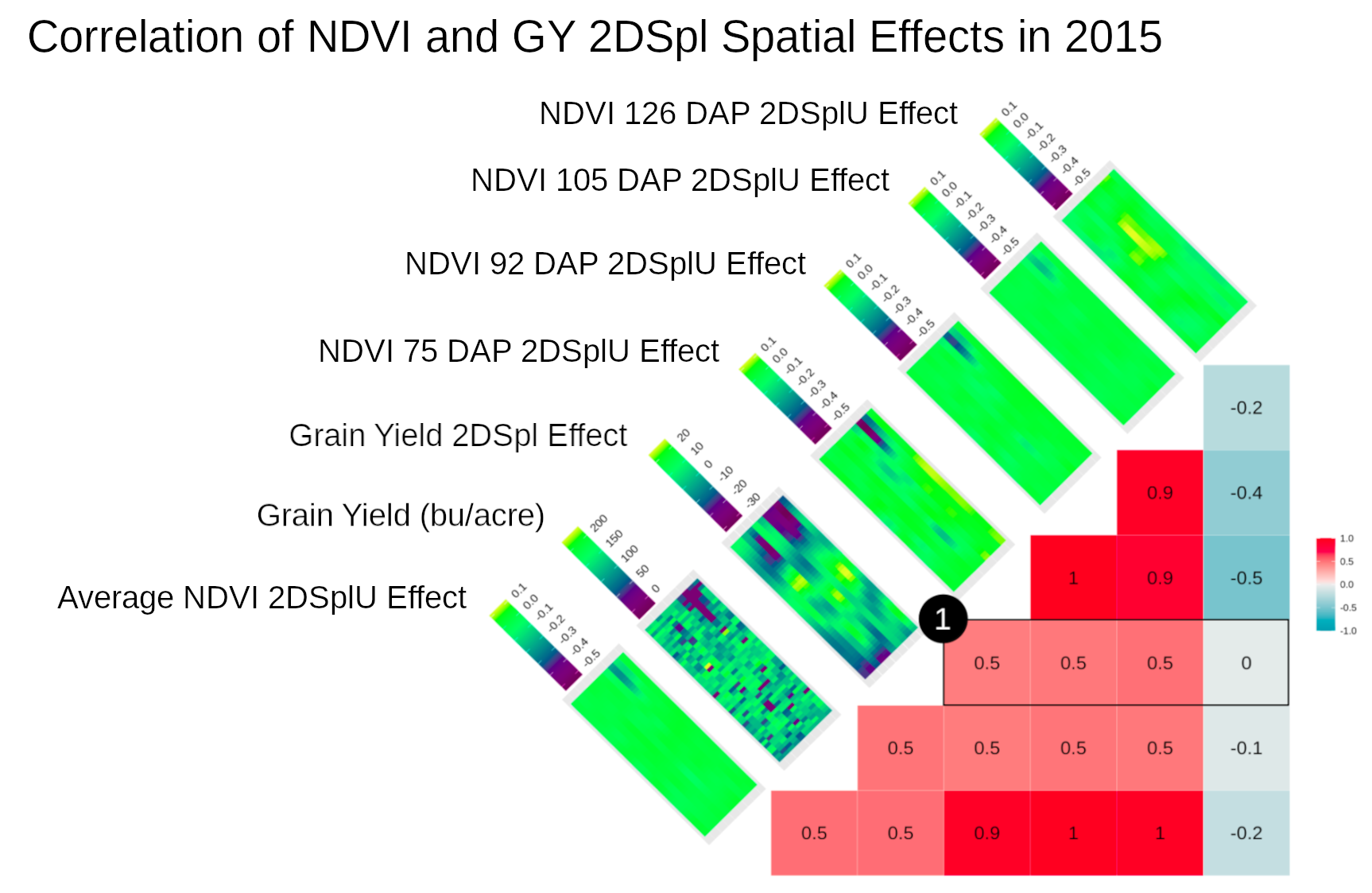


Figure S9: Shown are the two-dimensional spline (2DSpl) spatial effects for grain yield (GY) correlated with NDVI single-trait 2DSpl (2DSplU) permanent environment (PE) effects for 4 time points in the 2015_NYH2 experiment. As demonstrated in (1), correlations of 0.5 were observed at 75, 92, and 105 days after planting (DAP). The corresponding heatmaps drew the values over the rows and columns of the experimental plots, and consistently identified one poorly performing region in the field.


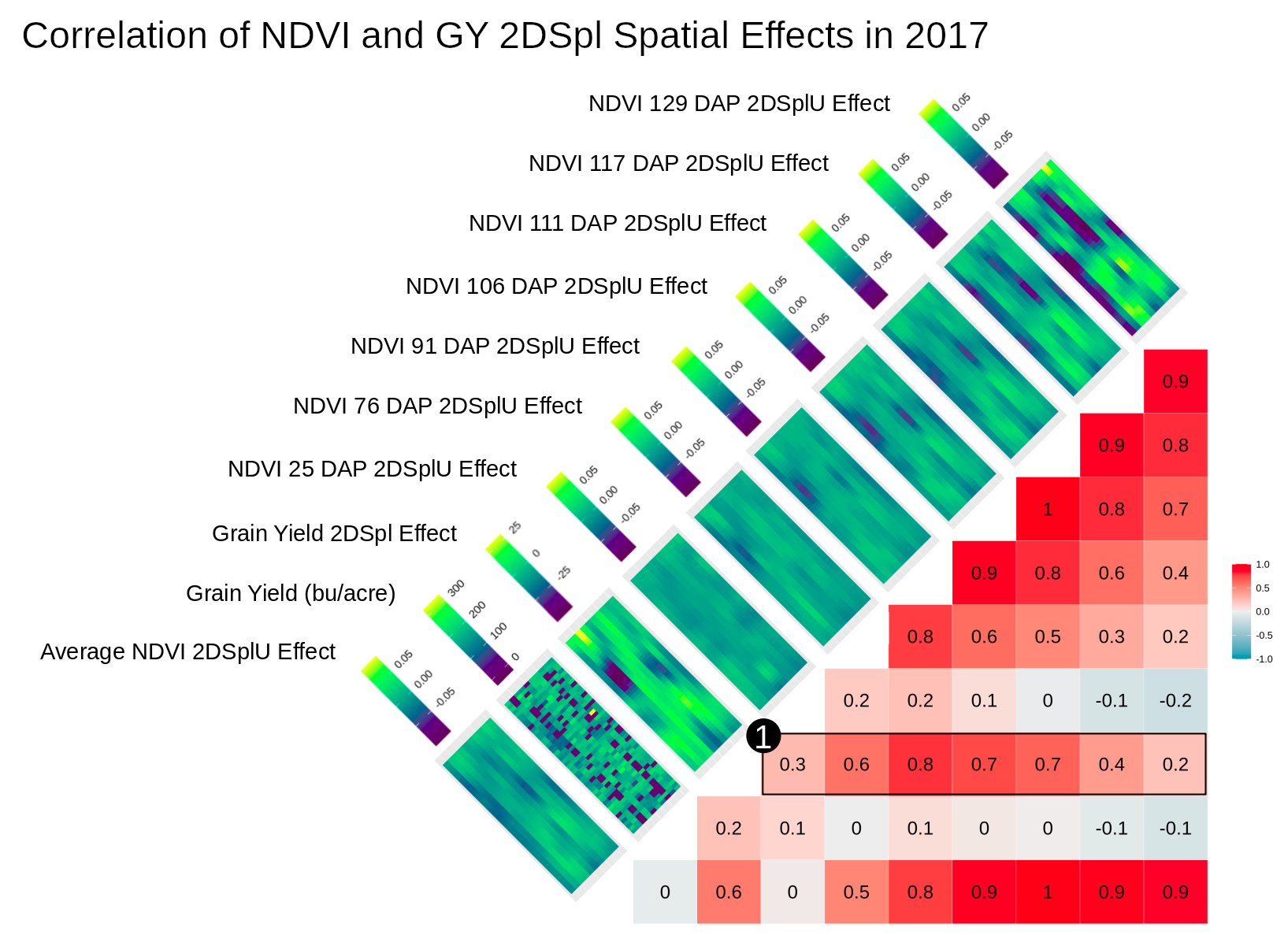


Figure S10: Shown are the two-dimensional spline (2DSpl) spatial effects for grain yield (GY) correlated with NDVI single-trait 2DSpl (2DSplU) permanent environment (PE) effects for 7 time points in the 2017_NYH2 experiment. As demonstrated in (1), correlations of 0.2 to 0.8 were observed with the highest correlation at 91 days after planting (DAP). The corresponding heatmaps drew the values over the rows and columns of the experimental plots, and consistently identified three distinct poorly performing regions in the field.


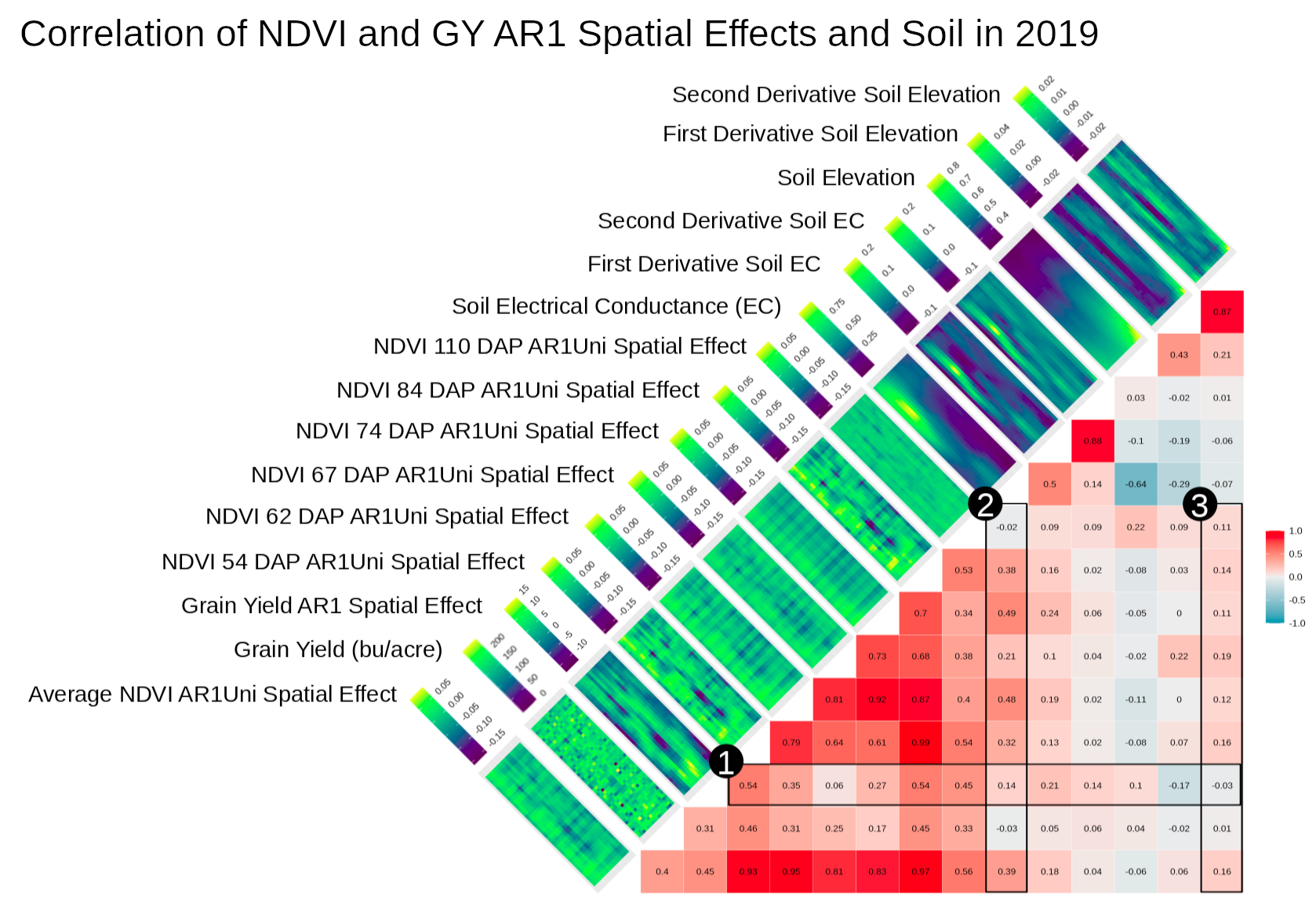


Figure S11: Drawn are the 2019_NYH2 AR1U NDVI PE observed over 6 time points correlated with GY and AR1 spatial effects of GY. Corresponding heatmaps showed values over the rows and columns of all experimental plots in the field, revealing similar spatial patterns. As indicated by (1), correlations of 0.1 to 0.5 between GY AR1 and the AR1U NDVI PE were found throughout the growing season. The highest correlation of 0.5 was observed at 110 DAP and 54 DAP. Soil EC and Alt, as well as the first and second numerical two-dimensional derivatives, were included with (2) showing correlations up to 0.5 for EC and (3) showing correlations up to 0.2 for d2Alt. The analog in Figure 3 showed that the 2DSplU NDVI PE were more highly correlated with the soil parameters than the AR1U NDVI PE, and the 2DSplU NDVI PE were more highly correlated with GY 2DSpl effects than the AR1U NDVI PE were correlated with the GY AR1 effects.


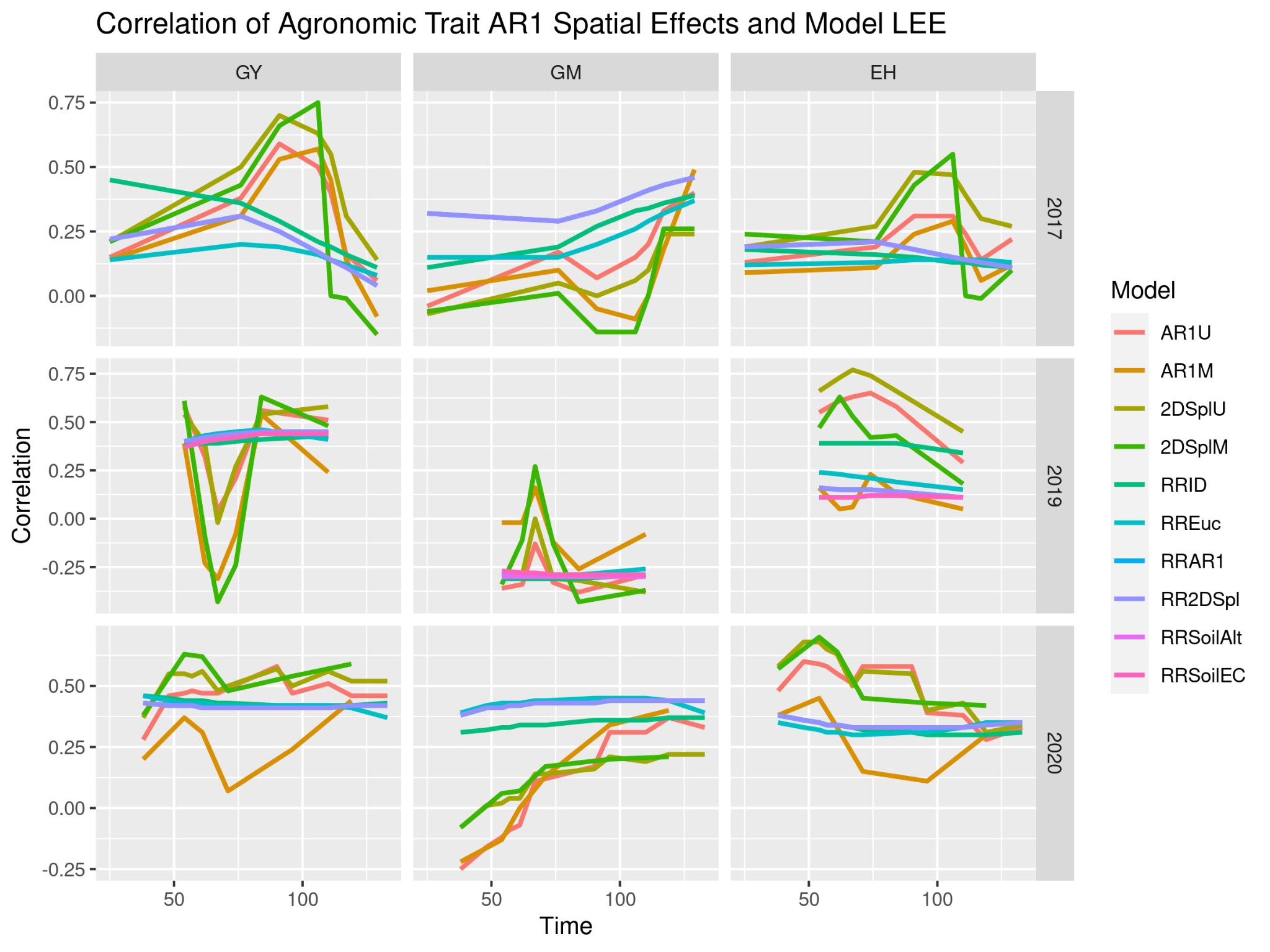


Figure S12: Illustrated are correlations between AR1 spatial effects of agronomic traits and NDVI PE from various models. Spatial effects for GY, GM, and EH in the 2017_NYH2, 2019_NYH2, and 2020_NYH2 field experiments were compared against NDVI PE across the growing season. Models run on traits in a given year showed similarities, with GY and EH following tandem trends, and GY and GM showing inverted patterns. 2015_NYH2 not included because convergence was not possible. The 2DSpl analog in Figure 4 illustrated similar patterns.


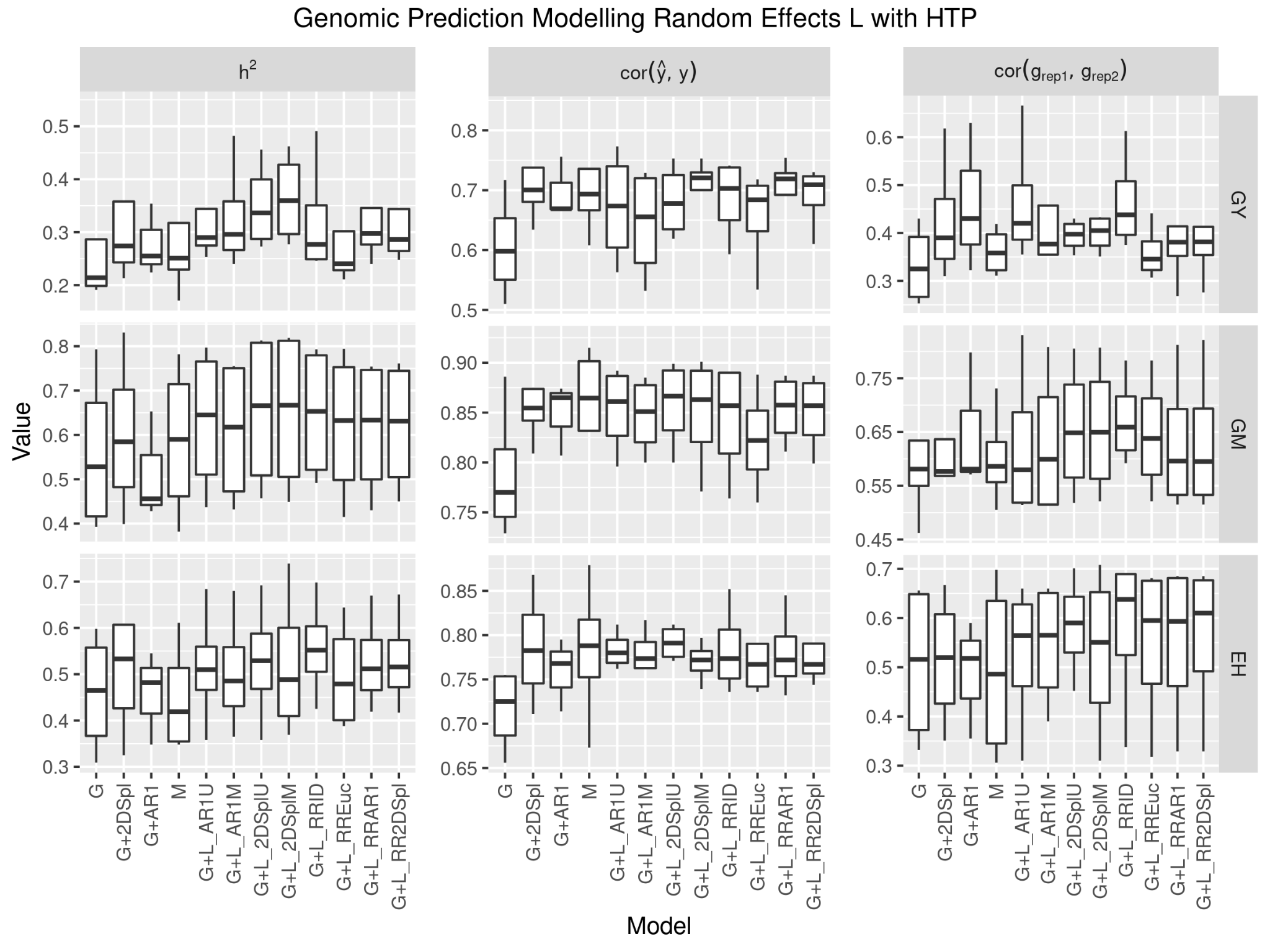


Figure S13: Genomic heritability, model fit, and genotypic effect estimation across replicates (GEER) in the four years for GY, GM, and EH, with NDVI PE implemented as L. The models G, G+2DSpl, and G+AR1 were baseline GBLUP and spatially corrected models, respectively, and M was a baseline multi-trait model. Models have L defined using NDVI PE of corresponding name.


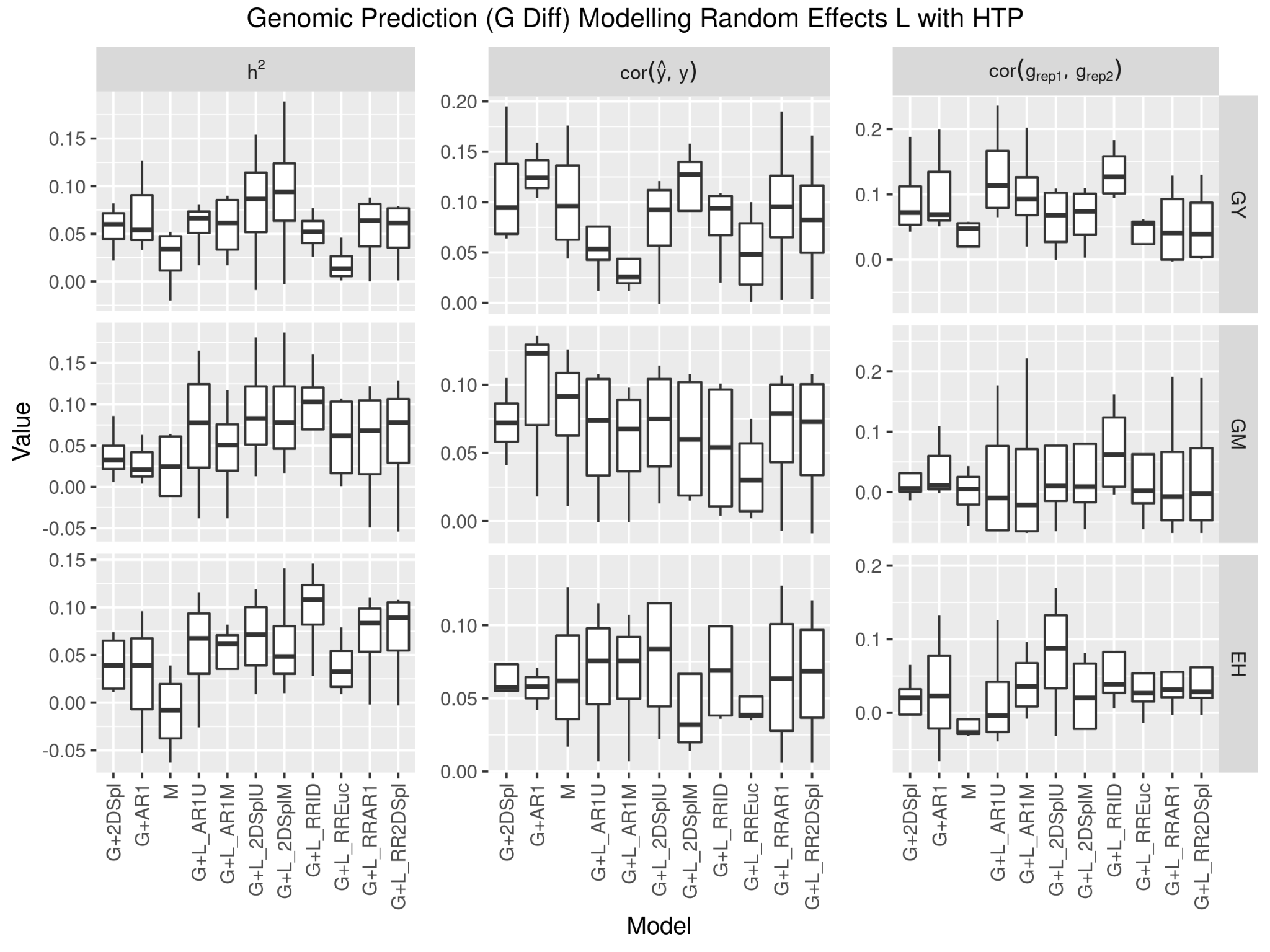


Figure S14: The difference in model genomic heritability, model fit, and genotypic effect estimation across replicates (GEER) with G (G Diff) in the four years for GY, GM, and EH, with NDVI PE implemented as L. The models G+2DSpl and G+AR1 were baseline spatially corrected GBLUP models, respectively, and M was a baseline multi-trait model. Models have L defined using NDVI PE of corresponding name.


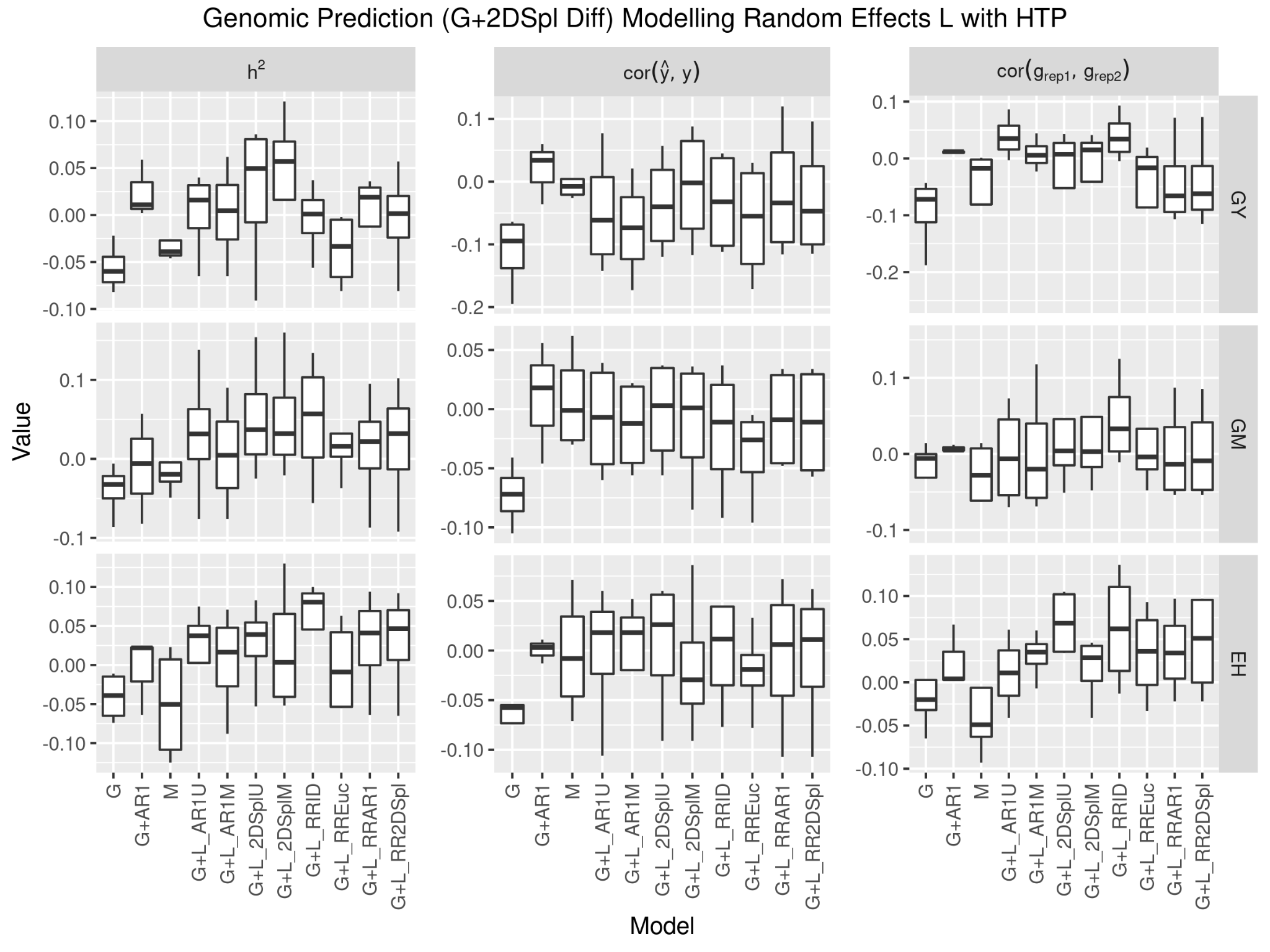


Figure S15: The difference in model genomic heritability, model fit, and genotypic effect estimation across replicates (GEER) with 2DSpl spatially corrected G (G+2DSpl Diff) in the four years for GY, GM, and EH, with NDVI PE implemented as L. The models G and G+AR1 were baseline GBLUP and spatially corrected models, respectively, and M was a baseline multi-trait model. Models have L defined using NDVI PE of corresponding name.


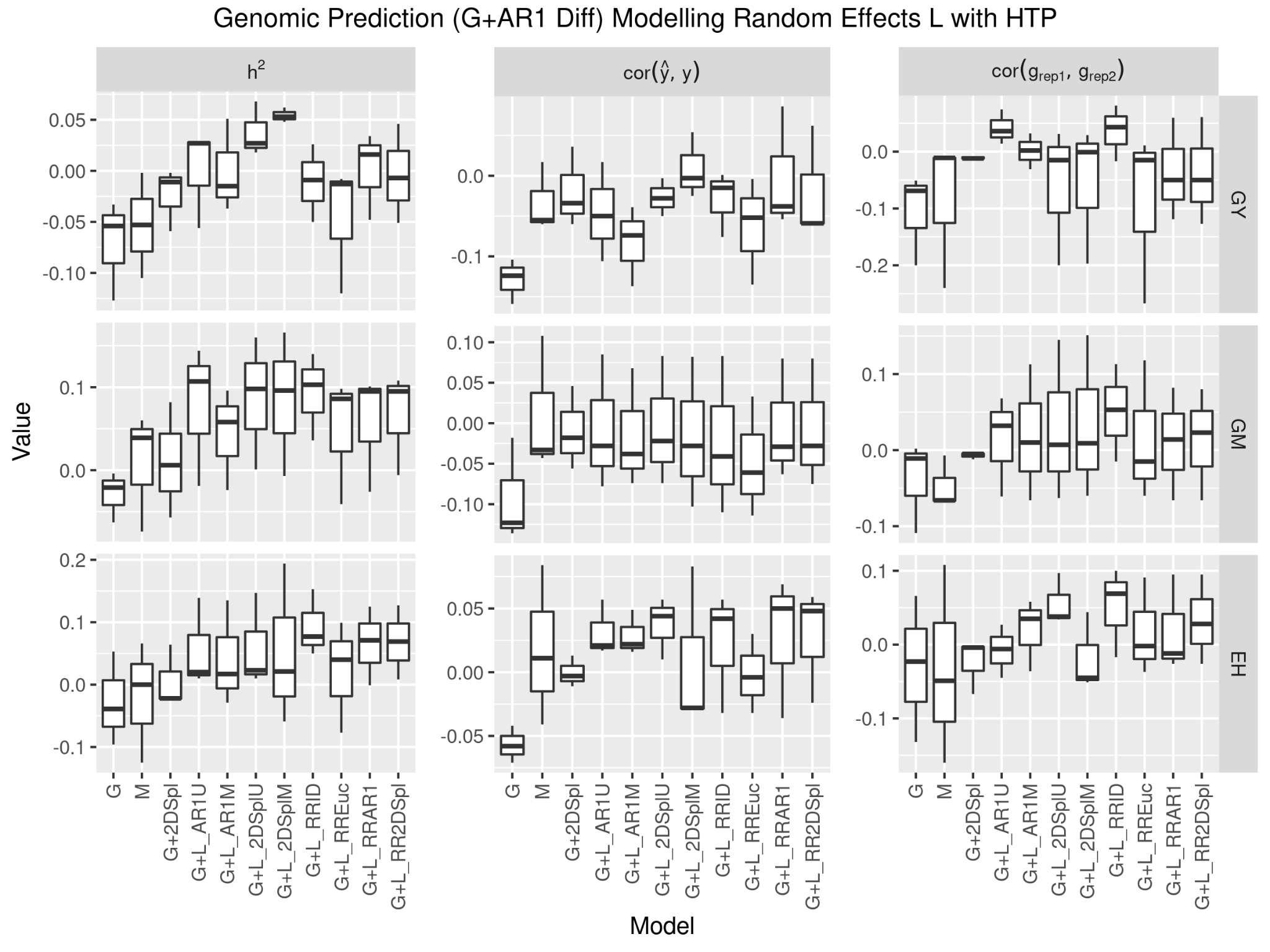


Figure S16: The difference in model genomic heritability, model fit, and genotypic effect estimation across replicates (GEER) with AR1 spatially corrected G (G+AR1 Diff) in the four years for GY, GM, and EH, with NDVI PE implemented as L. The models G and G+2DSpl were baseline GBLUP and spatially corrected models, respectively, and M was a multi-trait model. Models have L defined using NDVI PE of corresponding name.


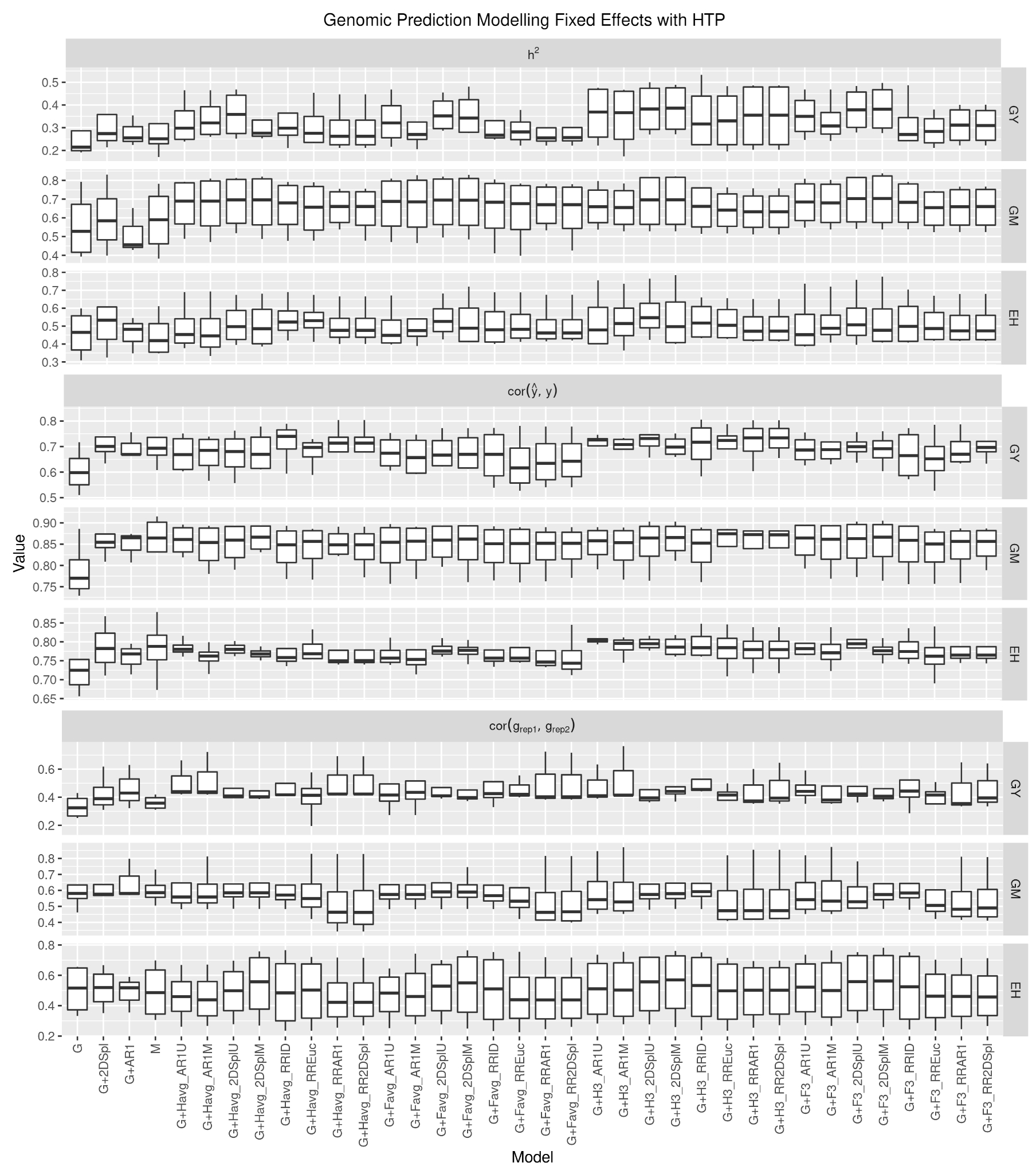


Figure S17: Genomic heritability, model fit, and genotypic effect estimation across replicates (GEER) in the four years for GY, GM, and EH, with NDVI PE implemented as FE. The models G, G+2DSpl, and G+AR1 were baseline GBLUP and spatially corrected models, respectively, and M was a baseline multi-trait model. Models have FE defined using NDVI PE of corresponding name.


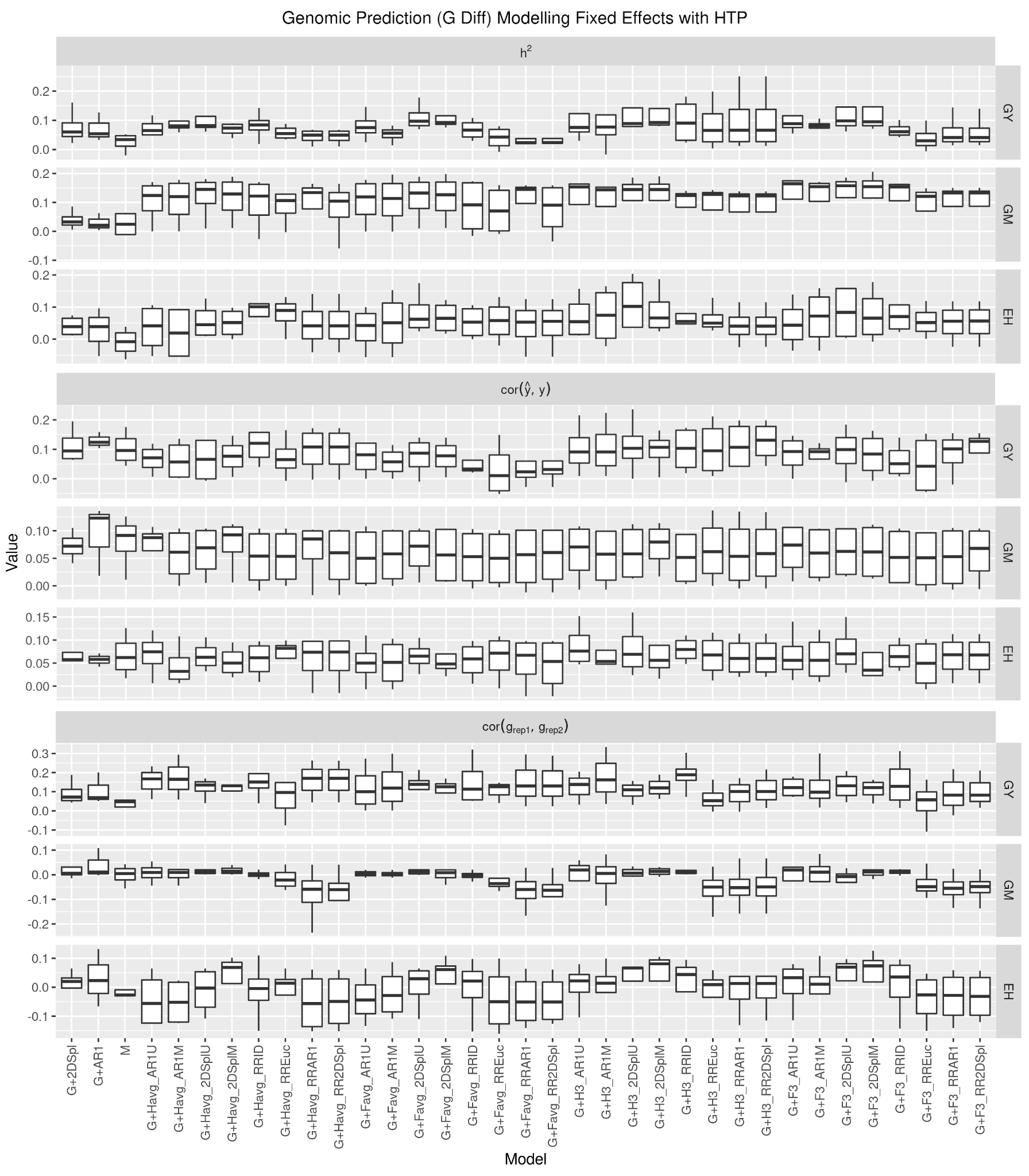


Figure S18: The difference in model genomic heritability, model fit, and genotypic effect estimation across replicates (GEER) with G (G Diff) in the four years for GY, GM, and EH, with NDVI PE implemented as FE. The models G+2DSpl and G+AR1 were baseline spatially corrected GBLUP models, respectively, and M was a baseline multi-trait model. Models have FE defined using NDVI PE of corresponding name.


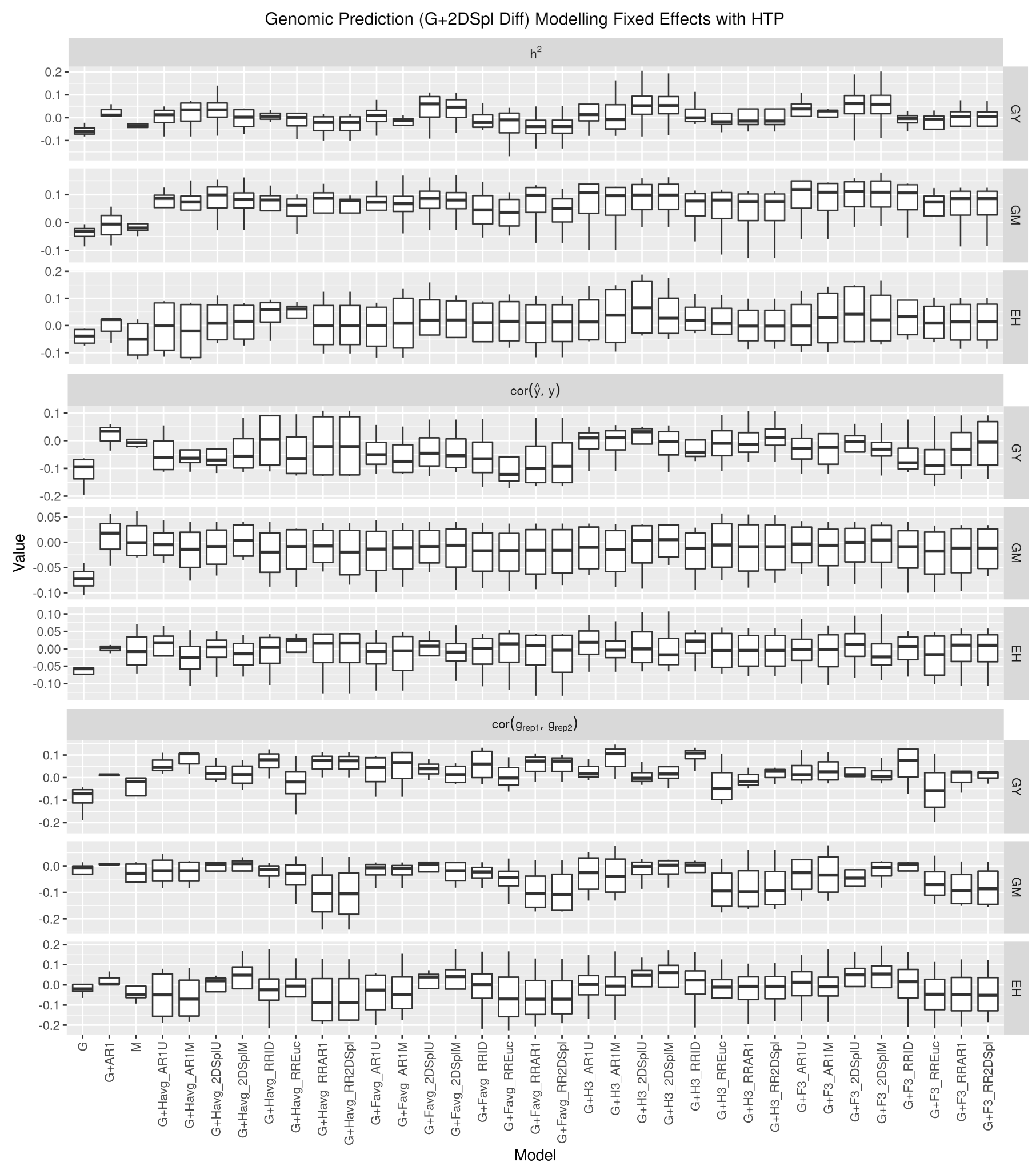


Figure S19: The difference in model genomic heritability, model fit, and genotypic effect estimation across replicates (GEER) with 2DSpl spatially corrected G (G+2DSpl Diff) in the four years for GY, GM, and EH, with NDVI PE implemented as FE. The models G and G+AR1 were baseline GBLUP and spatially corrected models, respectively, and M was a baseline multi-trait model. Models have FE defined using NDVI PE of corresponding name.


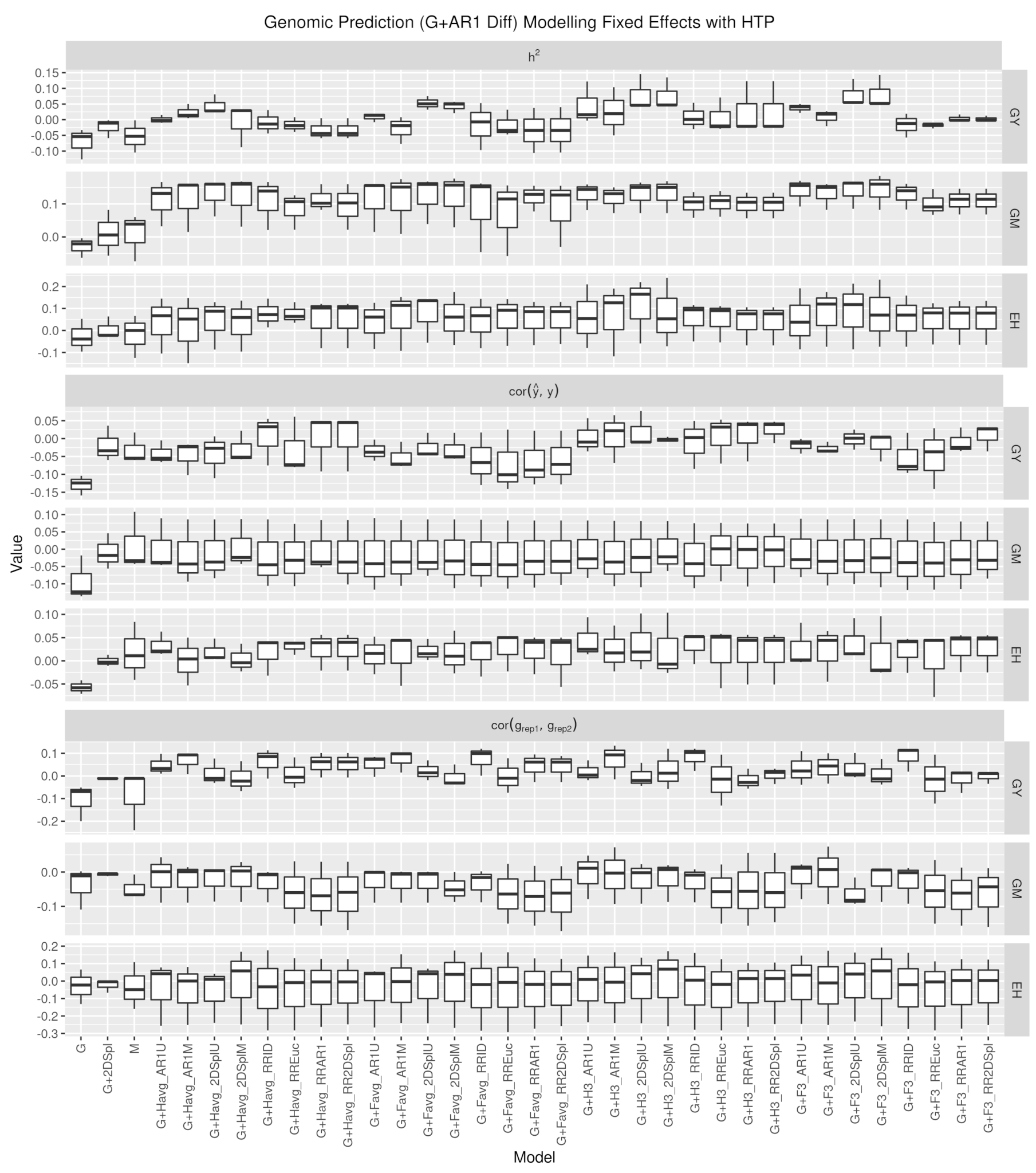


Figure S20: The difference in model genomic heritability, model fit, and genotypic effect estimation across replicates (GEER) with AR1 spatially corrected G (G+AR1 Diff) in the four years for GY, GM, and EH, with NDVI PE implemented as FE. The models G and G+2DSpl were baseline GBLUP and spatially corrected models, respectively, and M was a baseline multi-trait model. Models have FE defined using NDVI PE of corresponding name.


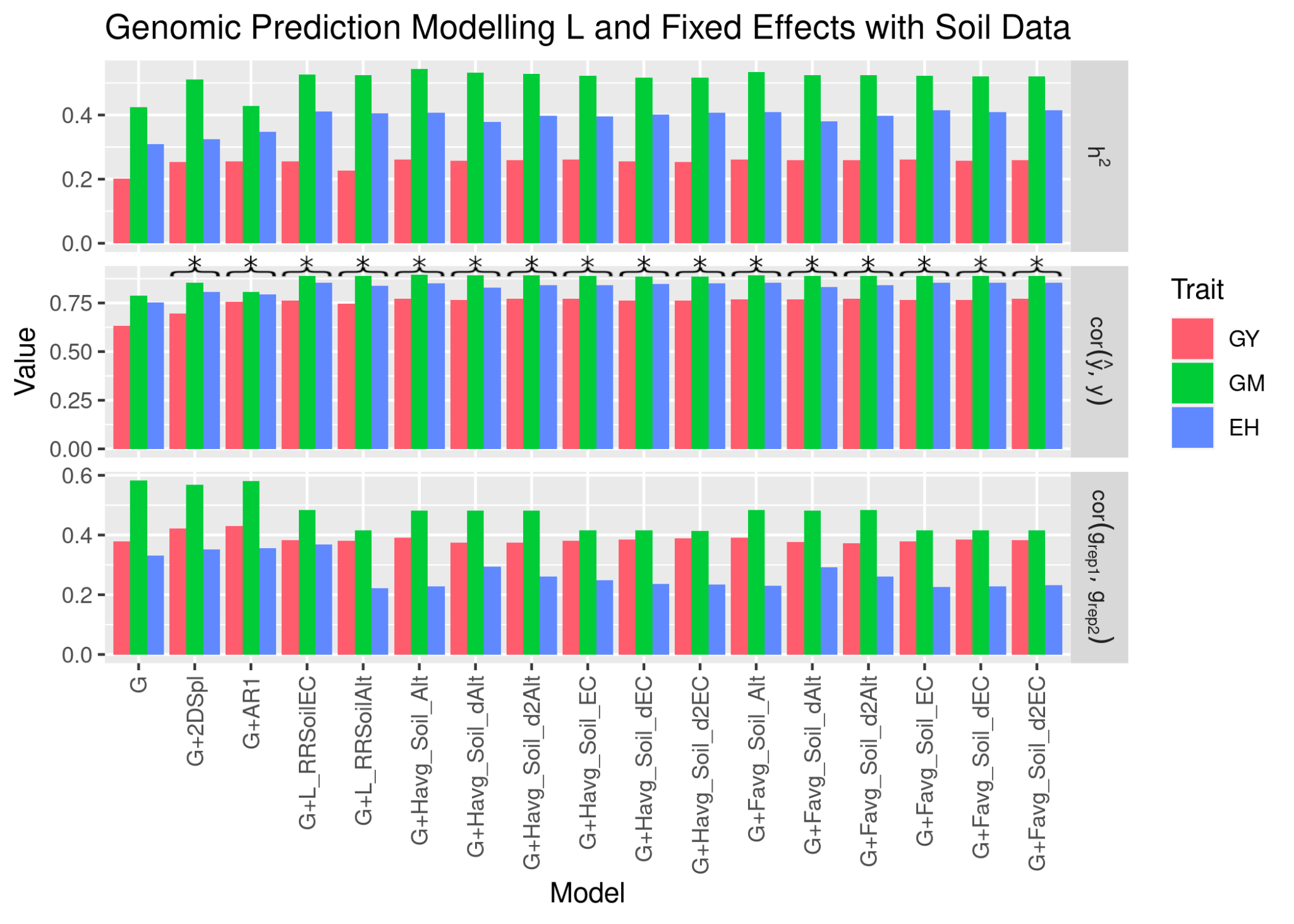


Figure S21: Genomic heritability, model fit, and genotypic effect estimation across replicates (GEER) in 2019_NYH2 for GY, GM, and EH, with NDVI PE implemented as L or as FE using soil data.


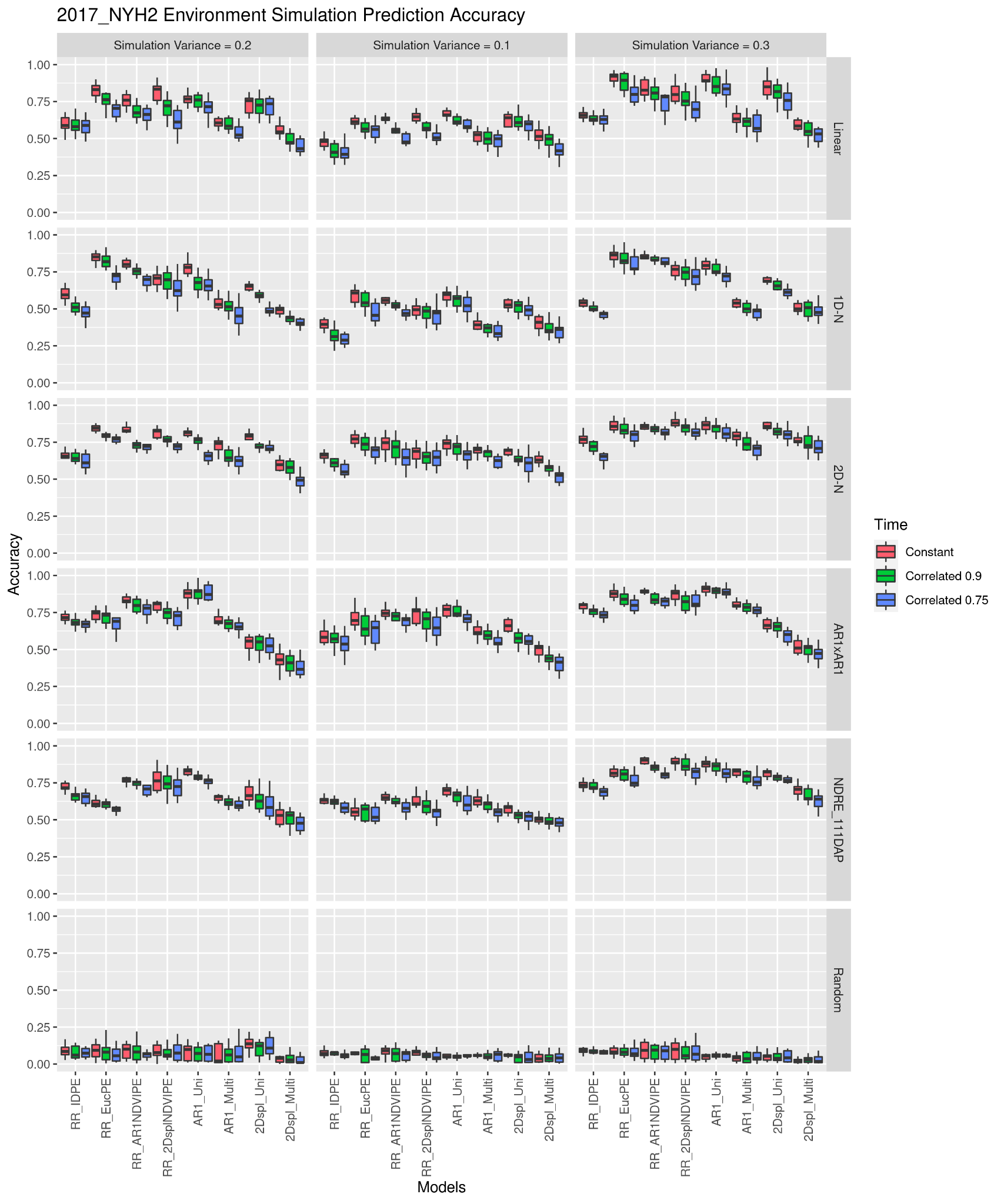


Figure S22: Prediction accuracy for six simulation processes (linear, 1D-N, 2D-N, AR1xAR1, random, and RD) were run ten times using the 2017_NYH2 NDVI values under a simulated environmental variance of 10%, 20%, and 30% and a simulated environmental effect that was 75%, 90%, and 100% correlated throughout the growing season. The RD scenario is illustrated as EC in this case. Prediction accuracy is the correlation of the simulated environmental effect and the model’s recovered effect.


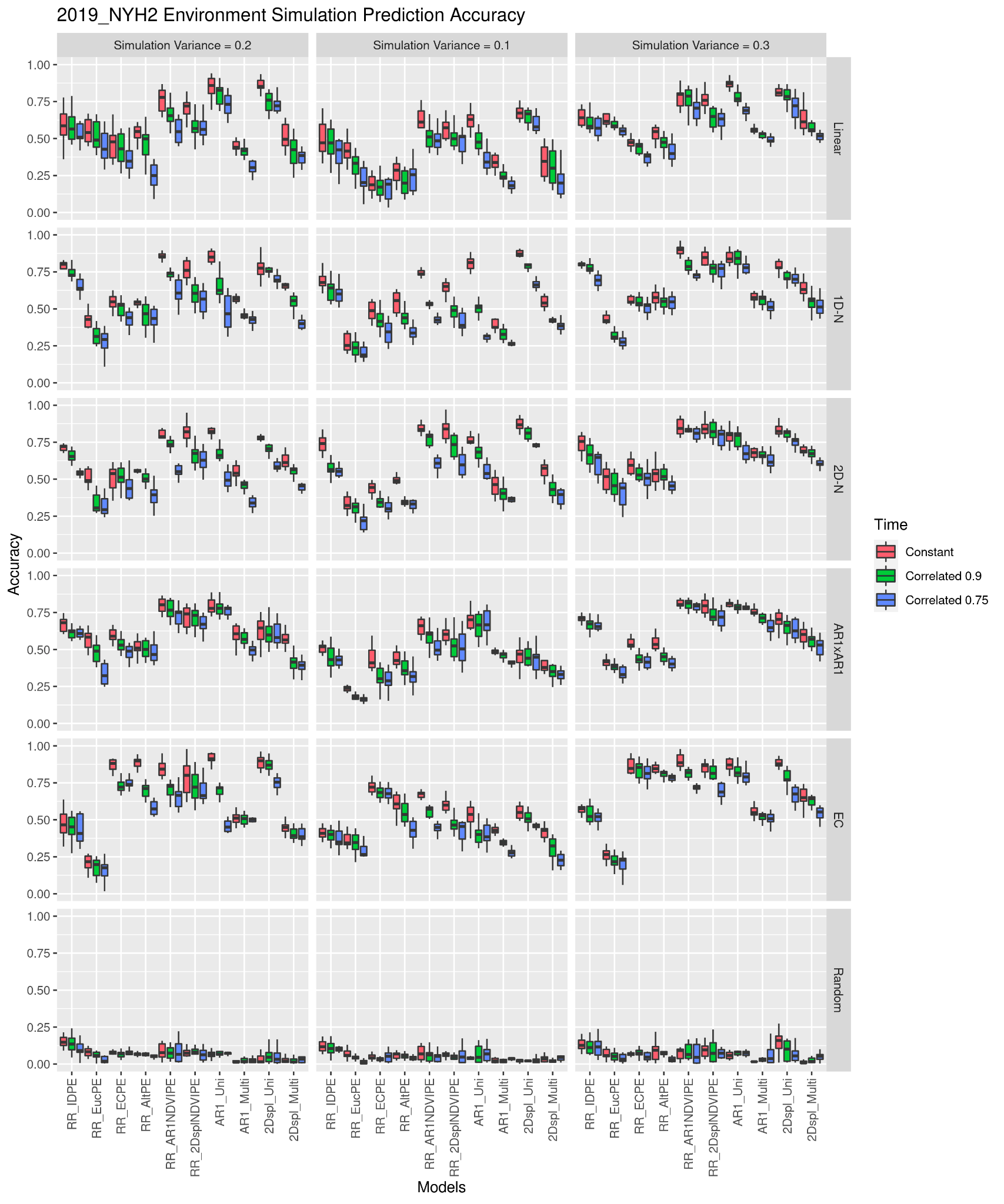


Figure S23: Prediction accuracy for six simulation processes (linear, 1D-N, 2D-N, AR1xAR1, random, and RD) were run ten times using the 2019_NYH2 NDVI values under a simulated environmental variance of 10%, 20%, and 30% and a simulated environmental effect that was 75%, 90%, and 100% correlated throughout the growing season. The RD scenario is illustrated as EC in this case. Prediction accuracy is the correlation of the simulated environmental effect and the model’s recovered effect.


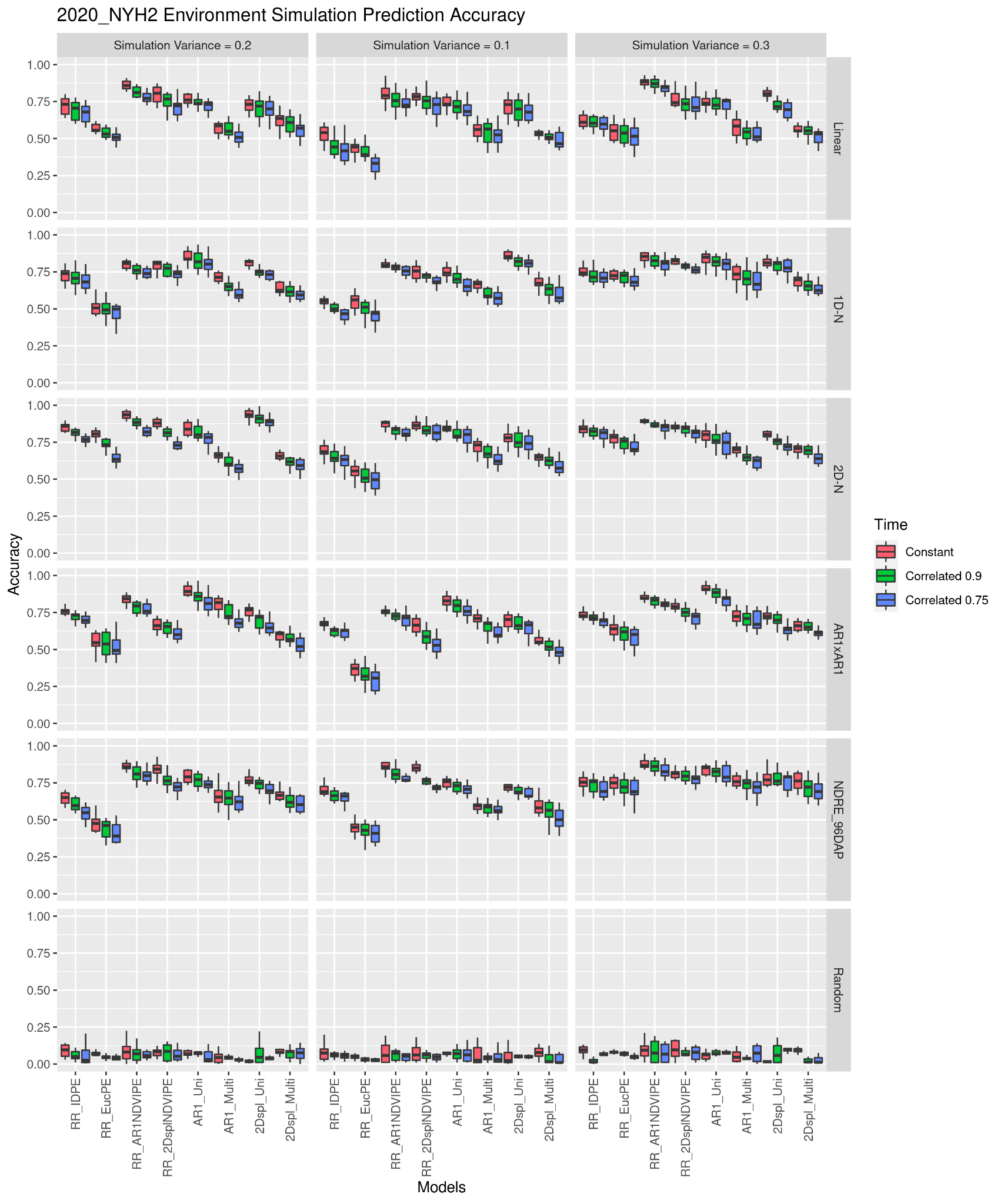


Figure S24: Prediction accuracy for six simulation processes (linear, 1D-N, 2D-N, AR1xAR1, random, and RD) were run ten times using the 2020_NYH2 NDVI values under a simulated environmental variance of 10%, 20%, and 30% and a simulated environmental effect that was 75%, 90%, and 100% correlated throughout the growing season. The RD scenario is illustrated as EC in this case. Prediction accuracy is the correlation of the simulated environmental effect and the model’s recovered effect.
